# Ubinuclein-1 confers histone H3.3-specific-binding by the HIRA histone chaperone complex

**DOI:** 10.1038/ncomms8711

**Published:** 2015-07-10

**Authors:** M Daniel Ricketts, Brian Frederick, Henry Hoff, Yong Tang, David C. Schultz, Taranjit Singh Rai, Maria Grazia Vizioli, Peter D. Adams, Ronen Marmorstein

**Affiliations:** 1 Department of Biochemistry and Biophysics, Perelman School of Medicine, University of Pennsylvania, Philadelphia, Pennsylvania 19104, USA; 2 Graduate Group in Biochemistry and Molecular Biophysics, Perelman School of Medicine, University of Pennsylvania, Philadelphia, Pennsylvania 19104, USA; 3 The Wistar Institute, Philadelphia, Pennsylvania, 19104, USA; 4 Institute of Cancer Sciences, CR-UK Beatson Labs, University of Glasgow, Glasgow G61 1BD, UK; 5 Institute of Biomedical and Environmental Health Research, University of West of Scotland, Paisley PA1 2BE, UK; 6 Abramson Family Cancer Research Institute, University of Pennsylvania, Philadelphia, Pennsylvania 19104, USA

## Abstract

Histone chaperones bind specific histones to mediate their storage, eviction or deposition from/or into chromatin. The HIRA histone chaperone complex, composed of HIRA, ubinuclein-1 (UBN1) and CABIN1, cooperates with the histone chaperone ASF1a to mediate H3.3-specific binding and chromatin deposition. Here we demonstrate that the conserved UBN1 Hpc2-related domain (HRD) is a novel H3.3-specific-binding domain. Biochemical and biophysical studies show the UBN1-HRD preferentially binds H3.3/H4 over H3.1/H4. X-ray crystallographic and mutational studies reveal that conserved residues within the UBN1-HRD and H3.3 G90 as key determinants of UBN1–H3.3-binding specificity. Comparison of the structure with the unrelated H3.3-specific chaperone DAXX reveals nearly identical points of contact between the chaperone and histone in the proximity of H3.3 G90, although the mechanism for H3.3 G90 recognition appears to be distinct. This study points to UBN1 as the determinant of H3.3-specific binding and deposition by the HIRA complex.

## Abstract (web summary)

Ubinuclein-1 (UBN1) is a subunit of the HIRA histone chaperone complex that deposits histone H3.3 into chromatin. Here the authors use structural and biochemical studies to show that a conserved domain in UBN1 mediates H3.3-specific binding by the HIRA complex.

## Graphical abstract

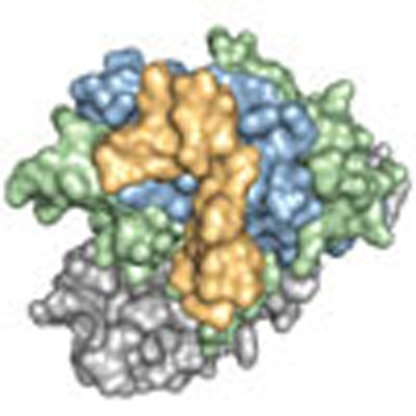

In metazoan cells, there are two major H3 variants: H3.1 is deposited by the trimeric CAF-1 complex during DNA replication^1^ and repair of ultraviolet-induced DNA damage^2^, while histone H3.3 is deposited in a replication-independent manner^1^,^3^ by either DAXX/ATRX largely at heterochromatin, including pericentromeres, telomeres and endogenous retroviral elements^4^,^5^,^6^,^7^, or by the HIRA complex predominantly at gene regulatory regions, gene bodies, developmentally regulated genes^4^,^8^,^9^,^10^,^11^,^12^,^13^, and sites of DNA and chromatin damage and repair^11^,^14^,^15^,^16^. However, DAXX/ATRX and HIRA likely also have some overlapping functions. For example, DAXX promotes H3.3 deposition at gene regulatory regions of activated neurons and both HIRA and DAXX are involved in H3.3 deposition in non-proliferating senescent cells^17^. While the H3/H4 histone chaperone ASF1a interacts with both CAF-1 and HIRA complexes and binds to both H3.3/H4 and H3.1/H4 for histone deposition^1^,^18^,^19^,^20^, CABIN1 and ubinuclein-1 (UBN1) are unique members of the HIRA complex^1^. CABIN1 and UBN1 were initially identified as a negative regulator for calcineurin signalling in T lymphocytes^21^ and as a ubiquitously expressed nuclear protein that interacts with cellular and viral transcription factors^22^, respectively, and later shown to be functional members of the HIRA histone chaperone complex^1^,^8^,^12^,^23^.

The human HIRA complex is orthologous to the *Saccharomyces cerevisiae* Hir histone chaperone complex, which regulates histone gene transcription^24^,^25^ and mediates replication-independent deposition of H3/H4 in yeast^26^. HIRA is homologous to Hir1 and Hir2 (ref. 27), CABIN1 is homologous to Hir3 (refs. 1, 23, 28) and UBN1 is homologous to Hpc2 with strong sequence conservation in the Hpc2-related domain (HRD)^8^,^28^. Although the UBN1-HRD was initially proposed to mediate association with the WD repeats of HIRA^8^, we more recently demonstrated that a less conserved region of UBN1 that resides sixty residues N terminal to the HRD is, in fact, responsible for HIRA-binding activity in UBN1; we named this domain the NHRD^29^ (Fig. 1a). These data in combination with the observations of others^28^ led us to the hypothesis that that the highly conserved UBN1-HRD may function to confer histone H3.3-binding specificity.

The molecular basis for how the HIRA complex selectively binds to H3.3, which differs from H3.1 by only 5 amino acids, is unknown. In this study, we report that H3.3-specific binding by the HIRA complex is mediated by the UBN1-HRD. We demonstrate that the UBN1-HRD specifically binds to H3.3/H4 over H3.1/H4 in a manner that is independent of the protein HIRA. Determination of a crystal structure containing a UBN1-HRD peptide bound to H3.3/H4/Asf1 and mutational studies aided us in identification of residues within the UBN1-HRD and H3.3 that contribute to the specificity and strength of the UBN1/H3.3 interaction. In addition, the UBN1/H3.3/H4/Asf1 crystal structure reveals striking structural similarity between UBN1/H3.3/H4 and DAXX/H3.3/H4. Together, our results demonstrate that the UBN1-HRD mediates H3.3-specific binding by the HIRA complex and exemplify the evolutionary power of the H3.3 surface in encouraging the disparate histone chaperones UBN1 and DAXX to adopt similar structures.

## Results

### A region of UBN1 containing the HRD specifically binds to H3.3

**Figure 1:**
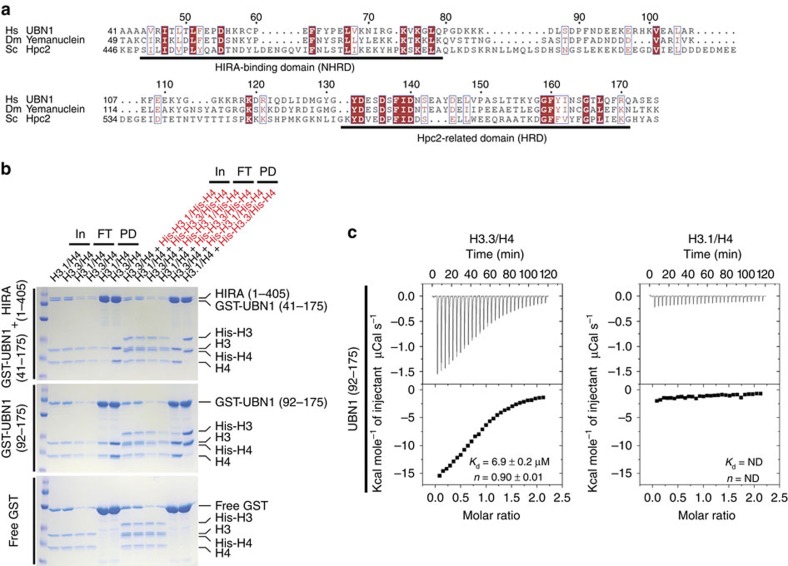
A region of UBN1 containing the Hpc2-related domain (HRD) is able to specifically bind to histone H3.3. (**a**) Sequence alignment comparing two highly conserved domains in *Homo sapiens* UBN1, *Drosophila melanogaster* Yemanuclein and *Saccharomyces cerevisiae* Hpc2. (**b**) GST pull-down histone-binding assay. Binding to H3.3/H4 and H3.1/H4 is tested separately in a single input H3/H4 pull-down (left), and in competition by subjecting an equimolar mixture of H3/H4 and His-H3/His-H4 to pull down (right). The H3.3-specific binding activity of GST-UBN1(41–175)/HIRA(1–405) (top), GST-UBN1(92–175) (middle) and free GST (bottom) is compared. (**c**) Isothermal titration calorimetry was used to quantitatively compare UBN1(92–175) binding with H3.3/H4 (left) and H3.1/H4 (right), ± values represent the standard error of the ITC fit using Origin 7.0. ND, not determined.

**Figure 2:**
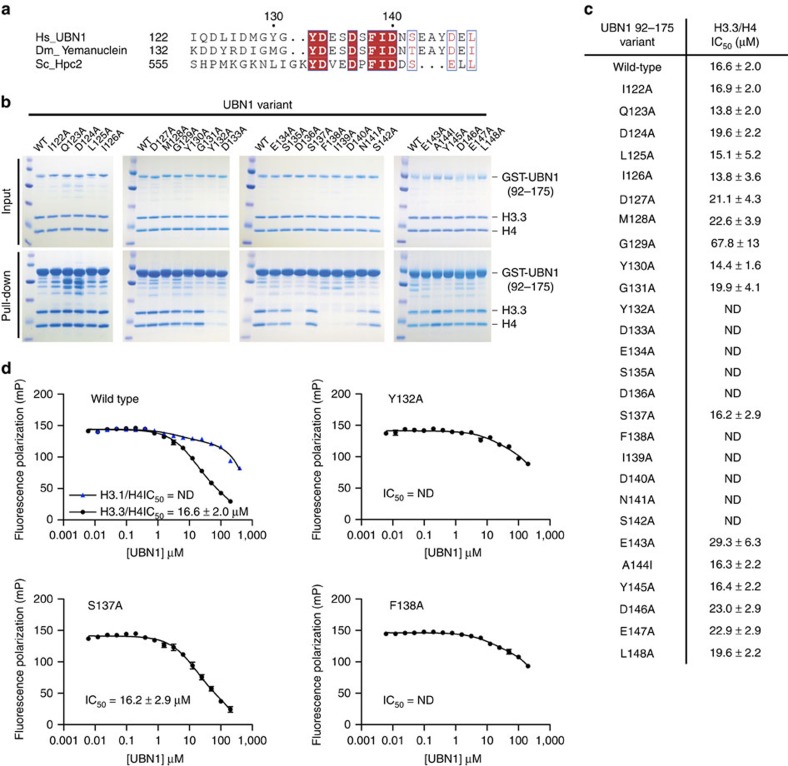
Conserved residues in the UBN1-HRD are required for binding with H3.3/H4. (**a**) Sequence alignment comparing HRD residues in *Homo sapiens* UBN1, *Drosophila melanogaster* Yemanuclein and *Saccharomyces cerevisiae* Hpc2, strictly conserved residues are highlighted in red. (**b**) GST pull-down assay comparing the H3.3-binding activity of UBN1 wild-type and 27 alanine scanning point mutants (A144 was mutated to I). (**c**) The IC_50_ for the binding with H3.3/H4 was quantified for each UBN1(92–175) variant using fluorescence polarization, ± values represent the standard error of the curves fit using GraphPad Prism 5.0a. (**d**) Four representative fluorescence polarization histone-binding curves are shown error bars represent the s.e.m. of three independent replicates. IC_50_, half-maximal inhibitory concentration; ND, not determined.

We initially compared H3.3/H4- and H3.1/H4-binding activity of the HIRA(1–405)/GST-UBN1(41–175) complex with that of GST-UBN1(92–175) (Fig. 1b). HIRA(1–405) has predicted WD repeats and is the minimum UBN1-binding domain, UBN(41–175) has both the NHRD and HRD while UBN1(92–175) harbours only the HRD. GST-tagged proteins were incubated with H3.1/H4 or H3.3/H4 alone or in competition with the reciprocal His-H3/His-H4 (H3.1 or H3.3) complex and subjected to GST pull-down. We observed H3.3/H4 selective binding over H3.1/H4 for both HIRA(1–405)/GST-UBN1(41–175) and GST-UBN (92–175), indicating that a UBN1-HRD containing protein region can specifically bind H3.3 without contribution from HIRA (Fig. 1b). In a control pull-down study, we demonstrated that GST-ASF1a binds to H3.1/H4 and H3.3/H4 with equal affinity, while GST-UBN1(92–175) specifically binds to H3.3/H4 (Supplementary Fig. 1). Specificity for H3.3/H4 over H3.1/H4 was especially marked when UBN1 was presented with both H3/H4 variant complexes simultaneously. Specificity for H3.3 was apparent in this assay regardless of whether H3.3 was His-tagged or untagged. Interaction of UBN1(92–175) with H3.3/H4 and H3.1/H4 was monitored directly with isothermal titration calorimetry (ITC) and indirectly through competition binding against a fluorescein isothiocyanate (FITC)-labelled UBN1 peptide (Fig. 1c; Fig. 2d; Supplementary Fig. 3a). Both assays demonstrated that UBN1(92–175) binds to H3.3/H4 with a dissociation constant in the low micromolar range (∼7–17 μM depending on the assay used), while significant interaction with H3.1/H4 could not be detected with either assay (Figs 1c and 2d).

### Conserved HRD residues are required for binding to H3.3/H4

UBN1 residues involved in the interaction with H3.3/H4 were investigated with an alanine scanning mutagenesis experiment targeted at the UBN1-HRD. A survey of the HRD was conducted by testing 18 GST-UBN1(92–175) triple alanine mutants in the context of GST-UBN1(92–175), spanning residues 122–175, for their ability to pull down with H3.3/H4. The strongest ablation of binding was observed for mutants of UBN1 residues 135–142 (Supplementary Fig. 2a,b), containing some of the most highly conserved residues of the HRD (Figs 1a and 2a). We then focused on this region by generating 27 alanine point mutations spanning UBN1 residues 122–148 (Fig. 2a). UBN1 point mutants were tested for interaction with H3.3/H4 using GST pull-down assays and fluorescence polarization histone-binding assays. Both experiments showed that the UBN1 Y132A, D133A, D136A, F138A, I139A, D140A and N141A mutants exhibited reduced histone binding relative to wild-type (WT) UBN1 (Fig. 2b–d; Supplementary Fig. 3). All of these residues are strictly conserved from yeast to human (Fig. 2a), with the exception of N141 (which is sometimes conservatively substitute to a D). Alanine mutants of UBN1 residues 122–148 were additionally subjected to pull down with H3.1/H4, and these mutants showed either no effect or a significantly smaller effect on UBN1 association with H3.1/H4, suggesting that the most conserved residues within the UBN1-HRD play a predominant role in H3.3-binding specificity (Supplementary Fig. 4).

**Figure 3:**
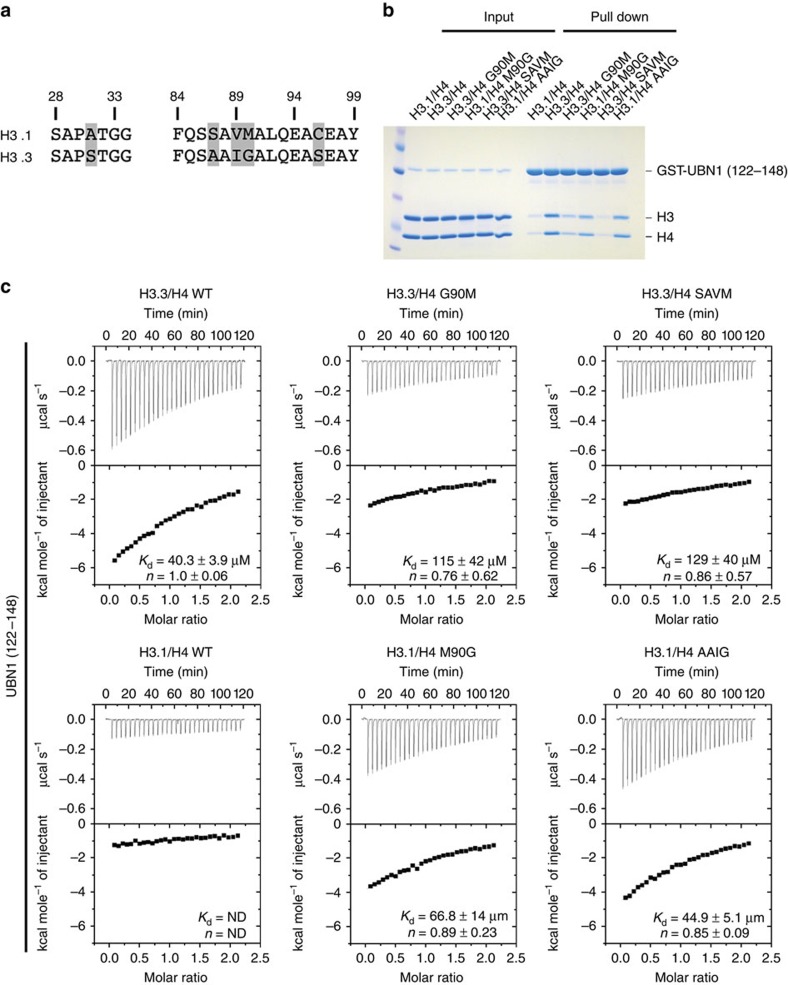
H3.3 residue G90 mediates UBN1 specificity. (**a**) Sequence alignment comparing human histones H3.1 and H3.3 with the five amino-acid differences highlighted in grey (**b**) GST pull-down assay comparing the ability of UBN1(122–148) to select between histone mutants where critical residues have been swapped between H3.1 and H3.3. UBN1 binding with wild-type H3.3/H4 and H3.1/H4 was analysed in addition to H3/H4 carrying several H3 mutants: H3.3(G90M), H3.1(M90G), H3.3(SAVM) and H3.1(AAIG). (**c**) Isothermal titration calorimetry quantification of the UBN1(122–148) interaction with wild-type and mutant H3/H4 complexes. Dissociation constant (*K*_d_) and stoichiometry of UBN1 binding to H3/H4 are reported, ±values represent the standard error of the ITC fit using Origin 7.0. ND, not determined.

A minimal UBN1-HRD fragment, consisting of residues 122–148, was then investigated to determine whether this conserved core region is sufficient for H3.3-binding specificity. In a pull-down assay GST-UBN1(122–148) exhibited specificity for binding to H3.3/H4 in comparison with H3.1/H4 (Fig. 3b). ITC and FP histone-binding assays were employed to quantitatively confirm the H3.3 specificity of the UBN1(122–148) peptide (Fig. 3c; Supplementary Fig. 5b). While UBN1(122–148) maintains binding specificity for H3.3/H4 in comparison with H3.1/H4, UBN1(92–175) binds to H3.3/H4 about three- to sixfold more strongly than UBN1(122–148). This suggests that residues flanking 122–148 of the UBN1-HRD also contribute to H3.3-binding affinity. UBN1(92–148) and UBN1(122–175) were analysed for binding to H3.3/H4 and H3.1/H4 with ITC to address the contributions of these flanking residues. We found that both UBN1 fragments bind H3.3/H4 with similar dissociation constants (*K*_d_=∼14–16 μM) while binding with H3.1/H4 was undetectable (Supplementary Fig. 5c). This binding capacity is stronger than UBN1(122–148) but weaker than UBN1(92–175), further suggesting that UBN1 residues 92–121 and 149–175 contribute to the binding affinity of H3.3/H4.

### H3.3 residue G90 mediates UBN1 specificity

To establish the amino acids in H3.3 and H3.1 that confer specific binding of the former to UBN1-HRD, we analysed the five residues that differ between H3.3 and H3.1 with a residue-swap mutagenesis experiment. The five differing residues are clustered in three patches along the sequence of H3 (S31A, SAVM87-90AAIG and C96S) (Fig. 3a; Supplementary Fig. 6a). We first tested H3.3(S31A), H3.3(SAVM) and H3.3(S96C) for binding to GST-UBN1(92–175) in pull-down assays, in competition with WT His-H3.1/His-H4 (Supplementary Fig. 6b). Only the H3.3(SAVM) mutant abolished H3.3-specific binding from UBN1 (Supplementary Fig. 6b). To assess the individual residue effects from this patch, H3.3 A87S, I89V and G90M mutants were applied in the same pull-down assays; only H3.3(G90M) prevented binding to UBN1 (Supplementary Fig. 6c). The reverse experiment, with H3.3 residues introduced into H3.1, showed that the H3.1(M90G) mutant uniquely acquired binding to UBN1 (Supplementary Fig. 6d). GST-ASF1a FL and GST-UBN1(92–175) were compared for selectivity between H3.3(SAVM) and H3.1(AAIG) mutants, and only GST-UBN1(92–175) exhibited binding specificity for H3.1(AAIG) (Supplementary Fig. 6e). GST-UBN1(122–148) was analysed for selectivity between the crucial histone residue-swap mutants and showed decreased binding to H3.3(SAVM) and H3.3(G90M), but increased binding to H3.1(AAIG) and H3.1(M90G) (Fig. 3b). A more quantitative analysis of the effect of H3.1 and H3.3 mutants on UBN1(122–148) binding was performed with ITC and demonstrated that the H3.3(G90M) and H3.3(SAVM) mutants had about a threefold decrease in UBN1(122–148) binding relative to H3.3 WT, and that the H3.1(M90G) and H3.1(AAIG) swap mutants bound UBN1(122–148) nearly as well as H3.3 WT (Fig. 3c).

### UBN1/H3.3/H4/Asf1 structure reveals mode of H3.3 binding

**Figure 4:**
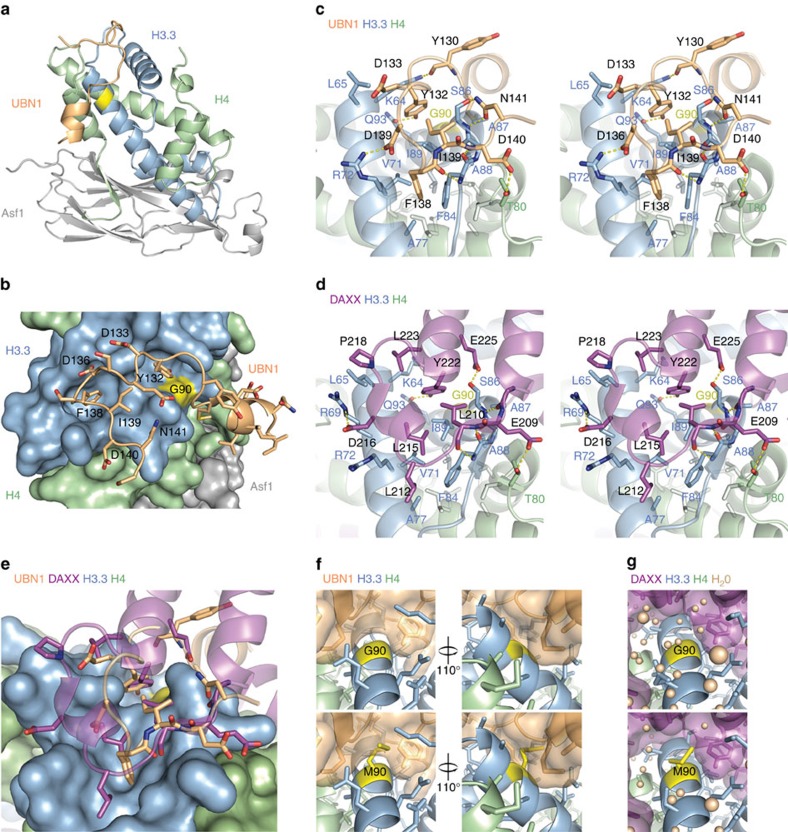
Crystal structure of the UBN1/H3.3/H4/Asf1 complex at 2.3-Å resolution. (**a**) Overall architecture of the UBN1/H3.3/H4/Asf1 complex with the position of H3.3 G90 highlighted in yellow. The structure is composed of UBN1 122–148, H3.3 60–133, H4 21–102 and Asf1 1–154. (**b**) UBN1 has an intimate association with the H3.3/H4 surface with close proximity to H3.3 G90 (yellow). UBN1 residues with sensitivity to alanine mutation are labelled. (**c**–**d**) Detailed stereo view of UBN1 and DAXX interactions with H3.3/H4 showing conserved interaction surfaces. (**e**) Alignment of the UBN1/H3.3/H4/Asf1 and DAXX/H3.3/H4 structures illustrating structural similarity between UBN1 and DAXX. (**f**) Surface representation of UBN1 shows that residues 128–132 rest closely over H3.3 G90 (top) and would likely sterically occlude binding with H3.1 M90 (bottom). M90 is represented by substitution of G90 with the highest percentage methionine rotamer. (**g**) The same view of the DAXX/H3.3/H4 structure shows that DAXX has a cavity where UBN1 128–131 resides. The comparable DAXX site fills with water molecules to sterically occlude binding with H3.1/H4.

Having determined that the UBN1-HRD is necessary and sufficient for selective binding of H3.3 over H3.1 to better understand this at the molecular level, we set out to determine a crystal structure of the UBN1/H3.3 interaction. We were unable to obtain crystals of UBN1 in complex with H3.3/H4 alone. Previously, a structure of the Asf1 core domain bound to H3.1/H4-lacking N-terminal tails was determined. We obtained this construct and modified it to generate a Asf1/H3.3/H4 complex that we successfully used for cocrystallization with a UBN1 peptide harbouring the HRD region. From those crystals, we determined the 2.3 Å X-ray crystal structure of a complex containing human UBN1(122–142) bound to *Xenopus laevis* H3.3(60–133), *X. laevis* H4(21–102) and *S. cerevisiae* Asf1(1–154) (Fig. 4a; Supplementary Table 1). Not surprisingly, the Asf1/H3.3/H4 component of the structure superimposes perfectly with the previously reported Asf1/H3.1/H4 structure (root mean squared deviation=0.821 Å for all identical atoms) since the differences between H3.3 and H3.1 do not reside at the Asf1–histone interface. An omit map confirms electron density for UBN1 residues 122–142 (Supplementary Fig. 7). UBN1 residues 122–127 form a short helix and residues 128–142 a coil, with the coil wrapping over a surface of H3.3 formed by helices 1 and 2 and the intervening turn. The UBN1 helix (residues 122–127) projects away into solvent (Fig. 4b). Confirming the biochemical studies, the UBN1-HRD makes intimate interactions with H3.3 centred around G90 (Fig. 4a,b). The UBN1–H3.3 interface is nucleated by UBN1 residues F138 and Y132 that protrude into largely hydrophobic pockets on H3.3 and position the coil of residues 128–132 in a saddle that sits over the top of H3.3 G90 (Fig. 4b). The hydroxyl group of UBN1 Y132 forms a hydrogen bond with the side chain of H3.3 Q93 (Fig. 4c). The aromatic ring of UBN1 Y132 makes extensive van der Waals interactions with the aliphatic side chain of H3.3 K64; a hydrogen bond with the UBN1 Y130 backbone carbonyl locks H3.3 K64 into place above UBN1 Y132 (Fig. 4c). A van der Waals interaction between the beta carbon of UBN1 D133 and the side chain of H3.3 L65 may also help anchor UBN1 Y132 into position (Fig. 4c). UBN1 F138 sits in a hydrophobic pocket where it forms an extensive network of van der Waals interactions with the aliphatic chain of H3.3 R72 and the aromatic ring of H3.3 F84, as well as supporting interactions with H3.3 V71, A77 and I89 (Fig. 4c). UBN1 I139 makes van der Waals interactions with the H3.3 I89 side chain and helps to cap the hydrophobic pocket in which UBN1 F138 binds (Fig. 4c). UBN1 D136 and D140 form hydrogen bonds with H3.3 R72 and H4 T80, respectively, while the UBN1 N141 side chain makes hydrogen-bonding interactions with both the backbone amide and the side chain hydroxyl of H3.3 S86 (Fig. 4c). The backbone carbonyl from UBN1 F138 and backbone amide from UBN1 D140 form hydrogen bonds with the backbone amide and carbonyl from H3.3 F84 to further solidify the UBN1/H3.3 interaction (Fig. 4c). Notably, the structure reveals that evolutionarily conserved UBN1 residues whose mutation disrupts H3.3 binding form hydrogen bond or van der Waals interaction with H3.3/H4 (Fig. 4c). UBN1 residues that are not sensitive to mutation (E134, S135 and S137) do not make contact with H3.3/H4 (Supplementary Fig. 8).

### Asf1 and histones tails do not alter UBN1/H3.3 interaction

To confirm the veracity of the interactions between UBN1 and H3.3/H4 observed in our crystal structure, we investigated how the presence of Asf1 in complex with H3/H4 affects the interaction between UBN1 and H3/H4. We conducted ITC to determine the affinity of UBN1(122–148) for complexes assembled with Asf1 and FL H3/H4 or H3(60–135)/H4(20–102) lacking the N-terminal tails (Supplementary Fig. 9). We found that UBN1(122–148) interacts with the Asf1/H3.3(FL)/H4(FL) complex with a dissociation constant (∼49 μM) comparable to that of UBN1(122–148) binding to H3.3/H4 alone (∼40–46 μM depending on the assay); no significant interaction between UBN1(122–148) and Asf1/H3.1(FL)/H4(FL) was detected (Supplementary Fig. 9a). We then tested UBN1 (122–148) binding with Asf1/H3.3(60–135)/H4(20–102). The dissociation constant determined for this interaction (∼110 μM) is roughly twofold weaker than for UBN1(122–148) binding to the Asf1/H3.3(FL)/H4(FL) complex, although specificity for H3.3/H4 over H3.1/H4 was still evident (Supplementary Fig. 9b). These data suggest that the presence of Asf1 in complex with the histones does not significantly influence the binding and specificity of the UBN1-HRD for H3.3/H4 over H3.1/H4.

### UBN1 and DAXX bind to H3.3/H4 with structural similarity

A comparison of the structure reported here with the DAXX/H3.3/H4 complex^30^ reveals that while the histone chaperones UBN1 and DAXX share no sequence conservation, they bind the region of H3.3/H4 surrounding G90 with striking similarity (Fig. 4c–e; Supplementary Table 2). The structural similarity between how UBN1 and DAXX bind H3.3/H4 is evident although the two chaperones have opposite orientations of N- to C-terminal sequence when bound to H3.3/H4 and no conservation when aligned in either orientation (Fig. 4e). DAXX residues Y222, L210 and E209 are in essentially the same positions and mediate the same interactions as UBN1 residues Y132, I139 and D140, respectively (Fig. 4c,d; Supplementary Fig. 10). In addition, L212 and L215 from DAXX maintain the same van der Waals interaction network that UBN1 F138 forms with H3.3 (Fig. 4c,d). Similar backbone interactions are also maintained between the DAXX and UBN1 chaperones with H3.3 (Fig. 4c,d). Supplementary Table 2 summarizes the contacts that UBN1 and DAXX make with key H3.3/H4 residues.

**Figure 5:**
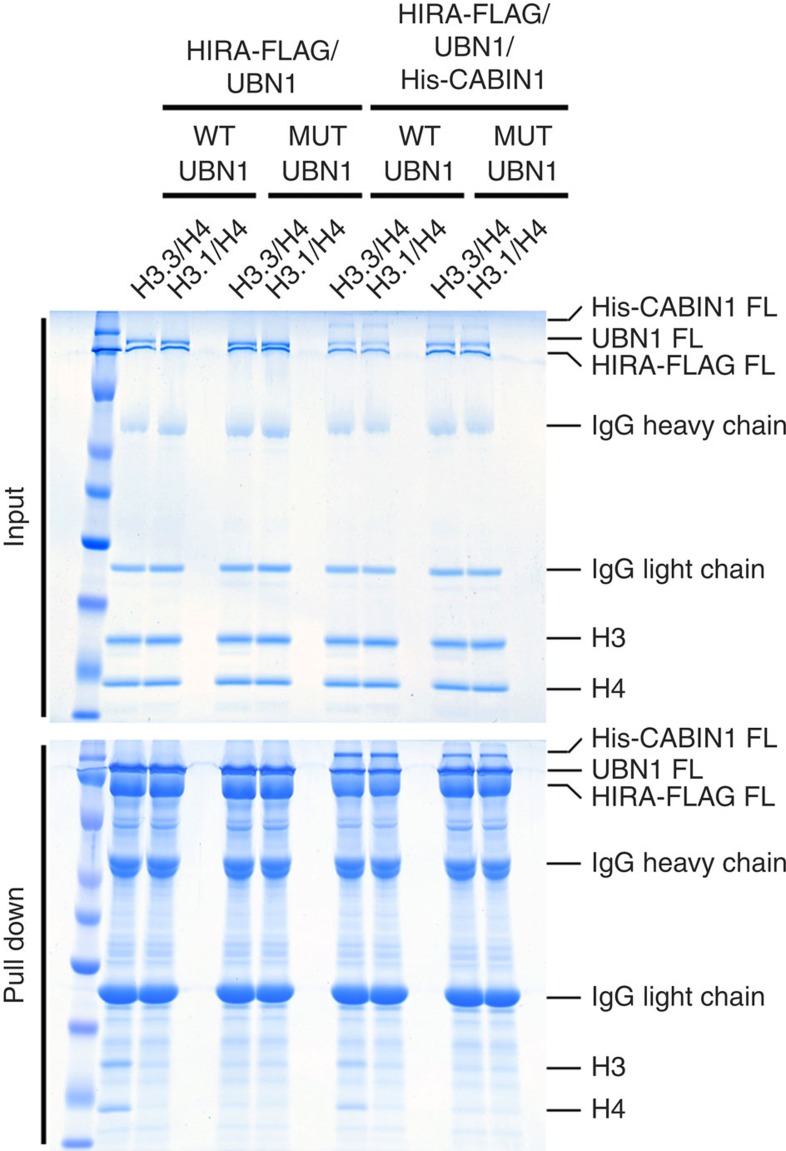
UBN1-HRD/H3.3 interface is required for H3.3-binding specificity in intact complexes. FLAG pull-down histone-binding assay. Recombinant complexes of HIRA-FLAG(FL)/UBN1(FL) and HIRA-FLAG(FL)/UBN1(FL)/His-CABIN1(FL) with wild-type and FID138-140AAA mutant UBN1 were analysed for binding to both H3.3/H4 and H3.1/H4.

While DAXX and UBN1 contact H3.3/H4 in much the same way, they do not use the same mechanism to select for H3.3 over H3.1. UBN1 residues M128-Y132 sit in close proximity above H3.3 G90 (Fig. 4b,f). A modelling of H3.3 G90M bound to UBN1 predicts that UBN1 would directly clash with a methionine at this position, suggesting that UBN1 employs a mechanism of steric occlusion to prevent unfavourable binding with H3.1 (Fig. 4f). In contrast, the corresponding region within the DAXX/H3.3/H4 complex is solvent accessible and filled with a network of water molecules that are proposed to provide steric bulk to occlude binding with H3.1 (ref. 24; Fig. 5g). Although G90 is the primary H3.3 residue responsible for UBN1 specificity, other H3.3-specific residues may contribute to the UBN1 interaction. The delta carbon from H3.3 I89 forms a van der Waals interaction with UBN1 I139 and the H3.3 A87 beta carbon forms van der Waals interactions with the beta and gamma carbons from UBN1 M128 (Fig. 5c). Both of these residues are unique to H3.3 and may be responsible for the increased affinity that UBN1 has for the SAVM(87–90)AAIG mutation in H3.1 in comparison with the individual M90G mutation (Fig. 3c). Taken together, while UBN1 and DAXX bind to the same region of histone H3.3, they achieve specificity for H3.3 over H3.1 by distinct mechanisms.

### H3.3-specific binding occurs in HIRA/UBN1/CABIN1 complex

To determine the contributions of the UBN1-HRD to H3.3 specificity in more complete HIRA and CABIN1-containing complexes, we prepared complexes containing full-length HIRA, UBN1 and CABIN1 proteins in WT form or bearing a UBN1 FID138-140AAA mutant, and assayed the ability of these complexes to pull-down H3.3/H4 and H3.1/H4. This experiment demonstrated that HIRA/UBN1 and HIRA/UBN1/CABIN1 complexes assembled from full-length proteins preferentially bind to H3.3/H4 over H3.1/H4, and the preference is abolished in complexes containing the UBN1 FID138-140AAA mutant (Fig. 5).

## Discussion

Our data demonstrate that the UBN1-HRD is an evolutionarily conserved H3.3-specific-binding domain that exploits residue G90 in H3.3 to mediate specific binding and so deposition by the HIRA histone chaperone complex. Many studies have shown that HIRA complex specifically binds to and deposits H3.3 and is required for deposition of H3.3 at selected genomic regions^1^,^4^,^7^,^9^,^11^,^17^,^31^. Our structural analysis shows that this specificity for H3.3 comes primarily from HIRA-bound UBN1, not HIRA itself.

The specificity of the UBN1-HRD for G90 may be evolutionarily conserved with the Hpc2 subunit of the yeast Hir complex. Recent data have shown that the yeast Hir complex can mediate H3.3-specific deposition when ectopically expressed in human cells and this specificity is dependent on the HRD in Hpc2 (ref. 32). In human cells, the UBN1-HRD functions to specifically bind H3.3 over H3.1 and presumably other variants containing M90 (H3.2, H3.4), and bulky L90 (CENP-A). H3.X, H3.Y and H3.5 represent primate-specific H3 variants that also contain G90 (refs 33, 34). Our data suggest that deposition of these H3 variants could also be coordinated by UBN1 (HIRA complex) or alternatively by DAXX.

We show that the structures of the UBN1 and DAXX interactions with H3.3 have surprising similarity, although the two chaperones use different mechanisms to mediate H3.3 G90 specificity. The structures are broadly similar in the sense that both chaperones bind proximal to H3.3 G90 of H3.3/H4 with nearly identical molecular contacts. However, unlike the UBN1-HRD, DAXX wraps around the entire H3/H4 heterodimer to make more extensive histone contacts that likely contribute to its enhanced general H3/H4-binding affinity relative to UBN1-HRD. Accordingly, it is possible that other UBN1 regions and/or other proteins within the HIRA complex also contribute to general histone-binding affinity^35^,^36^. In support of the participation of additional UBN1 regions in H3/H4 binding is our observation that UBN1(92–175) binds H3.3/H4 about three- to sixfold more strongly than UBN1(122–148) containing only the HRD (Supplementary Fig. 5b,c).

With respect to G90 specificity, the steric bulk provided by UBN1 residues 128–131 is perhaps more stringent than the water molecules that DAXX positions over G90. These water molecules can potentially be evacuated from the cavity to allow association with histone H3 variants with a bulkier amino acid at residue 90. This idea was demonstrated by a crystal structure of DAXX/H3.3(G90M)/H4 (ref. 30), and supported by a recent report^37^ that DAXX can mediate promiscuous deposition of the H3 variant CENP-A that contains an L in the corresponding position 90 of H3.

In addition, although we have demonstrated that UBN1 is primarily responsible for H3.3-binding specificity of the HIRA complex, the molecular basis for how the HIRA complex mediates deposition requires further studies. Our UBN1/H3.3/H4/Asf1 crystal structure may represent an intermediate in a potential hand-off of histones between ASF1a/H3.3/H4 and HIRA/UBN1/CABIN1 complexes before deposition into chromatin, as several studies suggest that deposition function of the HIRA complex is not dependent on the presence of ASF1a^14^,^38^,^39^. Nonetheless, the studies presented here provide important new molecular insights into how the HIRA histone chaperone complex specifically recognizes the histone H3.3 variant to regulate diverse genomic activities.

## Methods

### Multiple sequence alignments

Multiple sequence alignments for Figs 1a, 2a and 3a and Supplementary Figs 2a and 6a were generated using ClustalW2 (refs 40, 41). Alignments for Figs 1a and 2a and Supplementary Fig. 2a were further formatted for publication using ESPript version 3.0 to better visualize sequence conservation^42^. The alignment shown in Fig. 3a and Supplementary Fig. 6a was manually formatted for publication.

### Expression and purification of GST-tagged proteins

To generate the plasmid DNA construct encoding GST-UBN1(41–175), the complementary DNA (cDNA) encoding UBN1 residues 41–175 was PCR amplified from a previously described UBN1 construct^8^ and ligated into the BamHI/XhoI sites of pFastBacGST (Invitrogen). The plasmid DNA construct encoding HIRA(1–405) was generated by PCR amplification and ligation of the cDNA endoding HIRA(1–405) into the BamHI/XhoI sites of pFastBacHTB (Invitrogen), removal of the 6 × Histidine tag was facilitated by mutagenesis using the Stratagene QuikChange protocol^43^. GST-UBN1(41–175) and HIRA(1–405) were co-expressed in baculovirus-infected Sf9 cells. GST-ASF1a FL, GST-UBN1(92–175) and GST-UBN1(122–148) were generated through PCR by amplification of the proper cDNA sequence and ligation into the BamHI/XhoI sites of a custom engineered pCDFduet-1 (Novagen) *Escherichia coli* protein expression vector carrying an N-terminal GST tag that is removable through TEV protease cleavage. GST-UBN1(92–175) mutants were generated with the Stratagene QuikChange protocol^43^. GST-tagged proteins produced in *E. coli* were induced with 0.8 mM isopropyl-β-D-thiogalactoside (IPTG) added to BL21-Gold(DE3) cells (Agilent) containing the desired expression plasmid and cells were grown overnight at 18 °C. To purify GST-tagged proteins expressed in *E. coli* and Sf9, isolated cell pellets were suspended in 50 ml lysis buffer (PBS with 1 mM PMSF and 5 mM BME) per litre of cells and lysed with sonication. Lysate was clarified by centrifugation and the supernatant was incubated with glutathione agarose resin (Gold Biotechnology) for 1 h before washing the resin with 60 column volumes of wash buffer (PBS with 5 mM BME). GST-tagged proteins were either eluted from the glutathione agarose with five column volumes of elution buffer (PBS with 5 mM BME and 20 mM reduced glutathione) or TEV protease was added to the protein-bound glutathione agarose and incubated overnight to cleave the target protein off the GST fusion. Eluted GST-UBN1(92–175), GST-UBN1(122–148), GST-ASF1a and GST-UBN1(41–175)/HIRA(1–405) were then dialyzed overnight into buffer (20 mM Tris pH 8.0, 25 mM NaCl and 5 mM BME) for ion-exchange purification using a HiTrap Q HP column (GE Healthcare). Protein released from GST by TEV cleavage was eluted with six column volumes of buffer (HEPES pH 7.5, 750 mM and 1 mM TCEP) and concentrated using a 3 K cutoff centrifugal filter device (Millipore) before a final gel filtration purification step with a Superdex S75 10/300 GL column (GE Healthcare).

### Expression and purification of recombinant H3/H4

Human histones H3.3 and H3.2 were amplified by PCR from a human cDNA library (ATCC 77430) and ligated into the BamHI/XhoI sites of a custom engineered pETduet-1 (Novagen) *E. coli* protein expression vector with an N-terminal 6 × Histidine tag that is removable through TEV protease cleavage. A construct to express the amino-acid sequence for H3.1 was generated by site-directed mutagenesis of H3.2. Human histone H4 has a sequence that carries several rare codons, which are not conducive to expression in *E. coli*, and to overcome this issue, we obtained a previously described codon-optimized construct encoding H4 from *X. laevis*, which has the same amino-acid sequence as human H4 (ref. 44). The codon-optimized *X. laevis* H4 cDNA was PCR amplified and ligated into the BamHI/XhoI sites of the same *E. coli* protein expression vector as the H3 constructs. H3 and H4 proteins were expressed as inclusion bodies in BL21-Gold(DE3) cells (Agilent) induced with 0.8 mM IPTG, protein was expressed for 4 h at 37 °C. To purify H3 and H4, cells were lysed with sonication in 50 ml of lysis buffer (20 mM Tris pH 7.5, 500 mM NaCl and 5 mM BME) per litre of cells and lysate was clarified with centrifugation. Cell pellets containing insoluble H3 or H4 were then resuspended in denaturing buffer (20 mM Tris pH 7.5, 500 mM NaCl, 7.0 M GdHCl and 5 mM BME) and clarified with centrifugation. Denatured H3 or H4 were incubated with Ni-NTA agarose resin for 1 h before washing the resin with 60 column volumes of denaturing buffer. Proteins were either eluted from the Ni-NTA agarose with five column volumes of elution buffer (300 mM imidazole, 20 mM Tris pH 7.5, 500 mM NaCl, 7.0 M GdHCl and 5 mM BME). Eluted proteins were the dialyzed into water and lyophilized. Refolding was conducted through resuspending the lyophilized H3 and H4 in denaturing buffer and mixing the two histones at 1:1 before refolding by dialysis against two changes of non-denaturing buffer (20 mM Tris pH 7.5, 1.0 M NaCl, 5 mM BME and 1 mM EDTA) followed by dialysis into a low-salt buffer (20 mM Tris pH 7.5, 100 mM NaCl, 5 mM BME and 1 mM EDTA) before ion-exchange purification using a HiTrap SP HP column (GE Healthcare). The 6 × Histidine tags on H3 and H4 were either left on the protein or removed by treatment with TEV protease during the refolding reaction. Following ion exchange the refolded H3/H4 complexes were further purified with gel filtration using a Superdex 200 10/300 GL column (GE Healthcare). H3.3 and H3.1 residue-swap point mutants were generated using the Stratagene QuikChange protocol^43^.

### Synthetic peptides

Large scale peptide synthesis of UBN1(122–148) and FITC-UBN1(127–148) was commissioned by a commercial vendor (Genscript).

### GST pull-down histone-binding assay

GST-tagged UBN1 proteins and H3/H4 complexes were prepared as described above. The pull-down experiments represented in Fig. 1 and Supplementary Fig. 2 were conducted by incubating 2 μM GST-tagged protein with 2 μM H3/H4 (or 2 μM H3/H4 in competition with 2 μM His-H3/His-H4) in a buffer containing 20 mM HEPES pH 7.5, 500 mM NaCl and 1 mM TCEP at 4 °C for 30 min. Proteins were then subjected to pull down by incubation with glutathione agarose resin (Gold Biotechnology) for 30 min. Resin was washed with 120 column volumes of buffer (20 mM HEPES pH 7.5, 500 mM NaCl and 1 mM TCEP) before elution of bound proteins by boiling resin in SDS gel-loading buffer. The pull-down experiments represented in Figs 2 and 3 as well as Supplementary Figs 1, 4, 5 and 6 were conducted by incubating 2 μM GST-tagged protein with 4 μM H3/H4 (or 4 μM H3/H4 in competition with 4 μM His-H3/His-H4) in a buffer containing 20 mM HEPES pH 7.5, 750 mM NaCl and 1 mM TCEP at 4 °C for 30 min. Proteins were then subjected to pull down by incubation with glutathione agarose resin (Gold Biotechnology) for 30 min. Resin was washed with 120 column volumes of buffer (20 mM HEPES pH 7.5, 750 mM NaCl and 1 mM TCEP) before elution of bound proteins by boiling resin in SDS gel-loading buffer. Results of the pull-down assays were analysed though visualization of input and pull-down samples with SDS–polyacrylamide gel electrophoresis. Gels were stained using Coomassie Brilliant Blue G-250.

### Isothermal titration calorimetry

Quantitative analysis of the UBN1/histone interaction was conducted with a VP-ITC microcalorimeter (MicroCal). All proteins/peptides were prepared in a buffer with 20 mM HEPES pH 7.5, 750 mM NaCl and 1 mM TCEP before ITC analysis. Experiments for Figs 1c and 3c were conducted by injecting 0.450 mM UBN1 into a calorimetry cell containing 30 μM H3/H4, where UBN1 was injected in 7-μl increments every 3 min at 22 °C and ITC reference power set to 30. Experiments for Supplementary Fig. 5c were conducted with the same cell and syringe concentrations but UBN1 was injected in 10-μl increments every 3 min at 22 °C and ITC reference power was set to 20. Data were analysed using Origin version 7.0 and corrected for heat of mixing and dilution.

### Fluorescence polarization histone-binding assay

Quantitative analysis of the UBN1/histone interaction was conducted with a FP-based competition-binding assay; all data were collected using a PerkinElmer EnVision Xcite Multilabel plate reader. All proteins and peptides for this assay were prepared in a buffer containing 20 mM HEPES pH 7.5, 750 mM NaCl and 1 mM TCEP. We commissioned commercial synthesis of a UBN1(127–148) peptide with an N-terminal FITC moiety (Genscript). The *K*_d_ of FITC-UBN1(127–148) for binding to H3.3/H4 (*K*_d_=4.3±0.2 μM) and H3.1/H4 (*K*_d_=17.7±2.3 μM) was measured by monitoring the change in fluorescence polarization when increasing H3/H4 is titrated into a constant concentration of FITC-UBN1(127–148) (10 nM) (Supplementary Fig. 3a). The curves were fit using a one site—total binding model with GraphPad Prism (version 5.0a) (*Y*=*B*_max_ × *X*/(*K*_d_+*X*)+NS × *X*+background)s. We found that the FITC moiety or linker used to attach it to UBN1 artificially enhances the UBN1/histone interaction in comparison with both UBN1(92–175) and UBN1(122–148) (Figs 1c, 2c,d and 3c; Supplementary Figs 3a and 5b). Despite this, we were still able to use FITC-UBN1(127–148) for a competition-binding assay. To obtain a suitable fluorescence polarization range in the competition-binding assay, the concentration of H3/H4 was held at or slightly above the relative dissociation constant for FITC-UBN1(127–148). Increasing concentration of UBN1 competitor was titrated into solutions containing constant concentrations of FITC-UBN1(127–148) (10 nM) and either H3.3/H4 (6.5 μM) or H3.1/H4 (25 μM) to estimate half-maximal inhibitory concentration values by competition binding. UBN1 WT and all 27 point mutants were also titrated into a solution containing only the FITC-UBN1(127–148) to generate curves used to subtract background polarization, which is possibly caused by UBN1 self-association. Curves for D136A, S137A, F138A, I139A and D140A had a slightly different scale in comparison with others and were normalized to the average baseline; normalization did not alter data interpretation. Competition-binding curves were fit using the one site—total binding model with GraphPad Prism (version 5.0a). Recombinant H3/H4 are known to form a strong (H3/H4)_2_ homotetramer at concentrations above 1 μM^45^. Due to this H3/H4 conformation, two molecules of UBN1 are necessary to completely compete both FITC-UBN1 molecules away from the histone tetramer. Taking the binding stoichiometry of (UBN1)_2_/(H3/H4)_2_ into account, the concentration of UBN1 reported in the fluorescence polarization titration curves actually represents half of the molecular concentration of UBN1.

### Crystallization and structure determination

We obtained as a generous gift from Mair Churchill (University of Colorado School of Medicine) the GST-Asf1/H3.1/H4 polycistronic *E. coli* co-expression construct that was previously used to produce Asf1/H3.1/H4 for crystallization and structure determination^19^. We used mutagenesis^43^ to generate a construct encoding Asf1/H3.3/H4 for our purposes. The GST-Asf1/H3/H4 complex was expressed in BL21-Gold(DE3) cells (Agilent) induced with 0.8 mM IPTG, protein was expressed for 8 h at 28 °C. Cell pellets were resuspended in 50 ml of lysis buffer (20 mM Tris pH 8.0, 500 mM NaCl, 1 mM PMSF and 5 mM BME) per litre of cells and lysed with sonication. Lysate was clarified by centrifugation and the supernatant was incubated with glutathione agarose resin (Gold Biotechnology) for 1 h before washing the resin with 60 column volumes of wash buffer (20 mM Tris pH 8.0, 500 mM NaCl and 5 mM BME). The Asf1/H3/H4 complex was then released from the resin with on-column cleavage using prescission protease. The cleaved Asf1/H3/H4 complex was eluted with six column volumes of elution buffer (20 mM Tris pH 8.0, 500 mM NaCl and 1 mM TCEP). The free Asf1/H3/H4 complex was then concentrated using a 3 K cutoff centrifugal filter device (Millipore) before a final gel filtration purification step with a HiLoad 16/600 Superdex S75 pg column (GE Healthcare). The UBN1(122–148)/Asf1/H3.3/H4 complex was formed by incubating 0.2 mM Asf1/H3.3/H4 with 0.8 mM UBN1(122–148) in a buffer containing 20 mM Tris pH 8.0, 500 mM NaCl and 1 mM TCEP before crystallization. Hanging drops for vapour diffusion were formed by mixing two volumes of protein solution with one volume of crystallization solution (0.1 M Sodium Cacodylate pH 6.0, 8% polyethylene glycol 8 K and 0.2 M NaCl) and crystals appeared within 48 h. X-ray diffraction data were collected using an in-house Rigaku MicroMax-007HF microfocus rotating anode X-ray generator and a Saturn 944+ charge-coupled device detector. The diffraction data were indexed and scaled using HLK3000, and a Molecular Replacement solution was obtained using Phaser in the Phenix software suite with Asf1/H3.1/H4 (PDB ID, 2HUE) used as a search model. The solution was refined using phenix.refine in the Phenix software suite and model building was carried out using the molecular graphics program Coot. The solution model contains UBN1(122–142), H3.3(61–134), H4(24–102) and Asf1(1–154). Electron density was not observed for Asf1 residues 155–169, H3.3 residue 60 and H4 residues 19–23. Table 1 shows a summary of the refinement statistics. Simulated annealing *F*_o_*−F*_c_ omit map for the UBN1 peptide was generated using phenix.refine in the Phenix software suite. Figures for publication were generated using the program PyMOL. Structure comparison of UBN1 and DAXX were carried out using the published crystal structures of DAXX/H3.3/H4 (PDB ID, 4H9N) and DAXX/H3.3(G90M)/H4 (PDB ID, 4H9O)^30^.

### Assembly of Asf1/H3(FL)/H4(FL) complexes

As described previously^46^, expression and purification of the heterotrimeric Asf1(1–169)/H3(60–135)/H4(20–102) complex from *E. coli* yields a large excess of Asf1 not in complex with H3/H4 that can be separated from the Asf1/H3/H4 complex on a HiLoad 16/600 Superdex S75 pg column (GE Healthcare). We collected this excess Asf1 to be used for complex formation with either FL H3.3/H4 or FL H3.1/H4. Complexes were formed through mixing a twofold excess of Asf1 with H3.3/H4 or H3.1/H4 in a buffer containing 20 mM HEPES pH 7.5, 2.0 M NaCl and 1 mM TCEP, this mixture was then dialyzed overnight into a buffer containing 20 mM HEPES pH 7.5, 750 mM NaCl and 1 mM TCEP. The mixture was then injected onto a HiLoad 16/600 Superdex S75 pg column (GE Healthcare) to separate the Asf1/H3/H4 complex from the excess Asf1. The resulting Asf1/H3/H4 was used for ITC studies.

### pFastBacDual HIRA/UBN1

A C-terminally FLAG-tagged open reading frame of full-length human HIRA encoded by nucleotides 221–3,271 of NM_003325 were PCR amplified and subcloned into the XmaI and SphI restriction sites 3′ to the P10 promoter in pFastBacDual. Nucleotides 843–4,247 of NM_016936 encoding full-length human UBN1 were PCR amplified and subcloned into the SalI and XbaI restriction sites 3′ to the PH promoter in pFastBacDual. The FID(138–140)AAA mutation was introduced into UBN1 in the pFastBacDual vector by the Stratagene QuikChange protocol^43^.

### Expression of recombinant proteins in insect cells

Sequence-confirmed pFastBac transfer vectors containing sequences for human HIRA, UBN1 and CABIN1 were transformed into DH10Bac cells. Proper recombination of the HIRA, UBN1 and CABIN1 sequences into the baculovirus genome was determined by PCR and positive bacmid DNAs were transfected into Sf9 cells^47^. High-titer passage 1 (P1) virus stocks were recovered 120 h post-transfection. A high-titer P2 virus stock was generated by infecting Sf9 at an multiplicity of infection of ∼0.1, followed by incubation for 120 h. For productions, 1 × 10^6^ Sf9 cells per ml in Sf900-III medium (Invitrogen) were infected with virus at an multiplicity of infection of 1. Infected cells were harvested 48 h post-infection.

### Histone pull-downs with the intact HIRA/UBN1/CABIN1 complex

The HIRA-FLAG/UBN1 and HIRA-FLAG/UBN1/His-CABIN1 complexes containing WT UBN1 or UBN1(FID138-140AAA) mutant was purified by suspending isolated cell pellets in 50 ml lysis buffer (20 mM Tris pH 8.0, 500 mM NaCl, 1 mM PMSF, 1 mM BME and a complete EDTA-free protease inhibitor tablet (Roche)) per litre of cells, and lyses with sonication. Lysate was clarified by centrifugation and the supernatant was incubated with ANTI-FLAG M2 affinity gel (Sigma-Aldrich) for 2 h before washing the gel with 60 column volumes of wash buffer (20 mM Tris pH 8.0, 500 mM NaCl and 1 mM BME). The FLAG gel was bound to capacity for both the WT and mutant complexes. To conduct the FLAG pull-down assay, 50 μl of bound gel was incubated for 30 min with 4 μM H3/H4 in a buffer with 20 mM Tris pH 8.0, 750 mM NaCl and 1 mM BME. Then the gel was washed with 120 column volumes of buffer (20 mM Tris pH 8.0, 750 mM NaCl and 1 mM BME) before elution of bound proteins by boiling the gel in SDS gel-loading buffer. Results of the pull-down assays were analysed through visualization of input and pull-down samples with SDS–polyacrylamide gel electrophoresis. Gels were stained using Coomassie Brilliant Blue G-250.

## Additional information

**Accession codes:** Coordinates and structure factors for the Asf1/H3.3/H4/UBN1 complex have been deposited in the Protein Data Bank under accession code 4ZBJ.

**How to cite this article:** Ricketts, M.D. *et al*. Ubinuclein-1 confers histone H3.3-specific binding by the HIRA histone chaperone complex. *Nat. Commun.* 6:7711 doi: 10.1038/ncomms8711 (2015).

## Supplementary Material

Supplementary Information Supplementary Figures 1-10 and Supplementary Tables 1-2

## Figures and Tables

**Table 1:** X-ray data collection and refinement statistics.

Data collection*	
Space group	P3_2_2_1_
Cell dimensions	
*a, b, c* (Å)	90.024, 90.024, 120.734
*α*, *β*, *γ* (°)	90.00, 90.00, 120.00
Wavelength (Å)	1.5418
Resolution (Å)	25.41–2.25 (2.33–2.25)†
*R*_sym_	5.4 (44.0)
*I/σI*	26.4 (2.7)
Completeness (%)	99.1 (93.3)
Redundancy	6.0 (3.8)
	
**Refinement statistics**	
Resolution (Å)	25.41–2.25 (2.33–2.25)
No. reflections (total/unique)	725,259/26,431
*R*_work_/*R*_free_ (%)	20.82/23.48
No. of atoms	2866
Protein	2692
Ligand/ion	16
Water	158
	
B**-factors (Å^2^)	
Protein (Asf1/H3.3/H4)	54.7
Protein (UBN1)	67.2
Ligand/ion	58.6
Water	53.4
	
R.m.s.d	
Bong lengths (Å)	0.003
Bond angles (°)	0.637
	
Ramachandran statistics	
Favoured (%)	99.1
Allowed (%)	0.90

r.m.s.d, root mean squared deviation

^*^One crystal was used for data collection and refinement.

^†^Values in parentheses are for the highest resolution shell.
