# Supplementary material for: Ubinuclein-1 confers histone H3.3-specific-binding by the HIRA histone chaperone complex
Source: Nat Commun. 2015 Jul 10;6:7711. doi: 10.1038/ncomms8711 (PMC4510971; doi:10.1038/ncomms8711)
Supplement: Supplementary Information — Supplementary Figures 1-10 and Supplementary Tables 1-2 [file ncomms8711-s1.pdf]

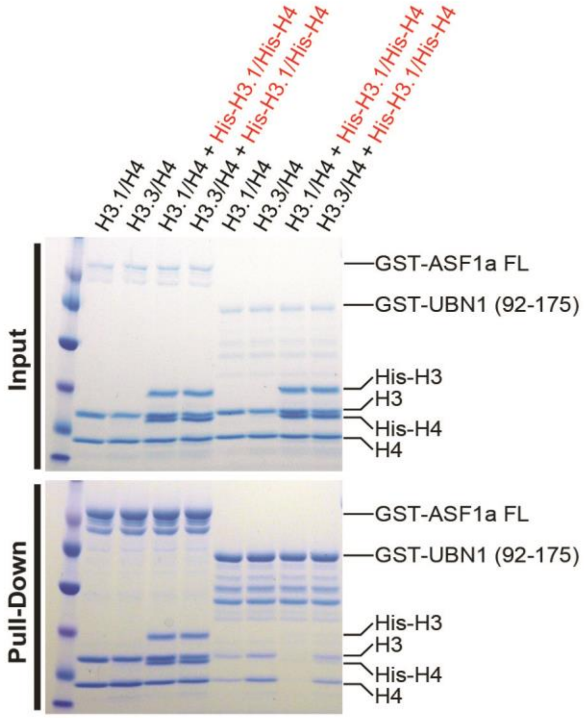

Supplementary Figure 1. ASF1a control pull-down. UBN1(92-175) (right) is able to selectively bind H3.3/H4 while ASF1a FL (left) is not. Both chaperones are tested for binding to H3.1/H3 and H3.3/H4 alone as well as in competition with His-H3.1/His-H4.

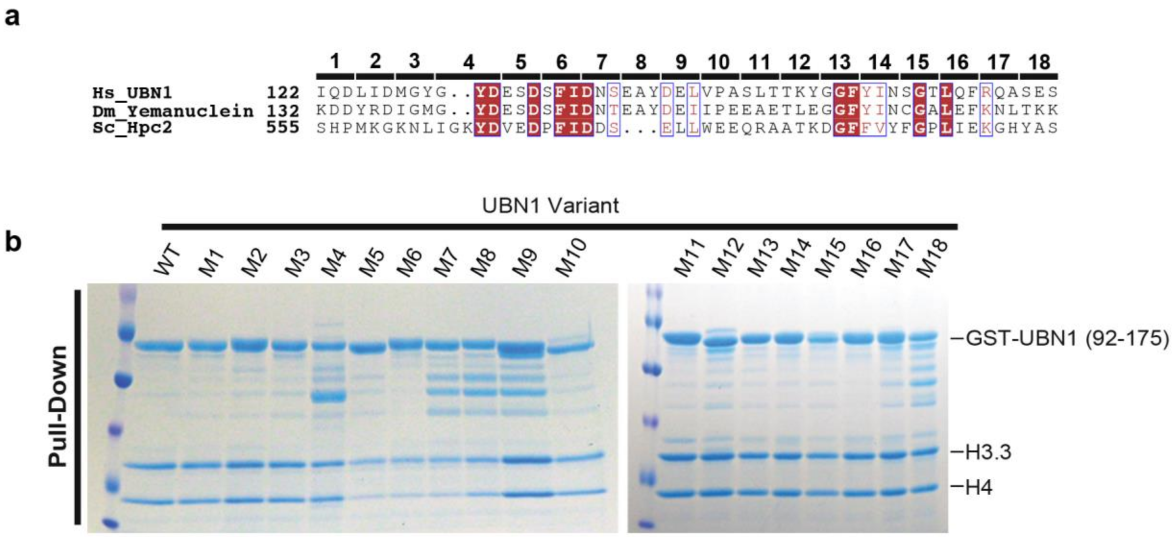

Supplementary Figure 2. Scanning alanine mutagenesis targeted at the UBN1 HRD. (a) A group of 18 triple alanine mutants were designed across the UBN1 HRD. (b) The gel representing a GST-pull down comparing the H3.3/H4 binding ability of the mutants to wild-type UBN1.

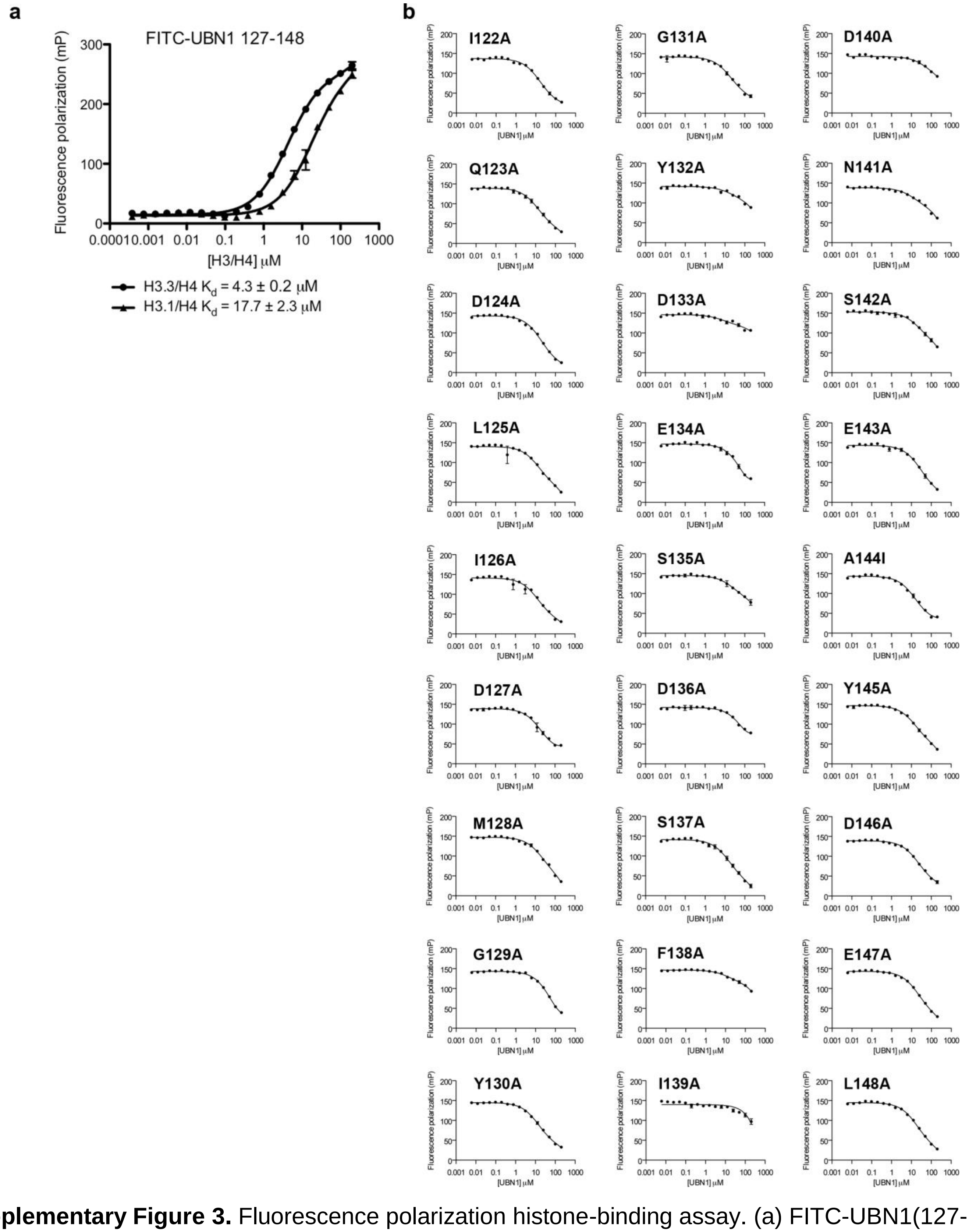

Supplementary Figure 3. Fluorescence polarization histone-binding assay. (a) FITC-UBN1(127-148) fluorescence polarization binding curves with both H3.3/H3 and H3.1/H4. (b) A group of 27 alanine point mutants of UBN1 (92-175) were analyzed for binding to H3.3/H4 with a competition binding fluorescence polarization assay. Error bars represent the s.e.m. of three independent replicates, +/- values represent the standard error of the curves fit using GraphPad Prism 5.0a

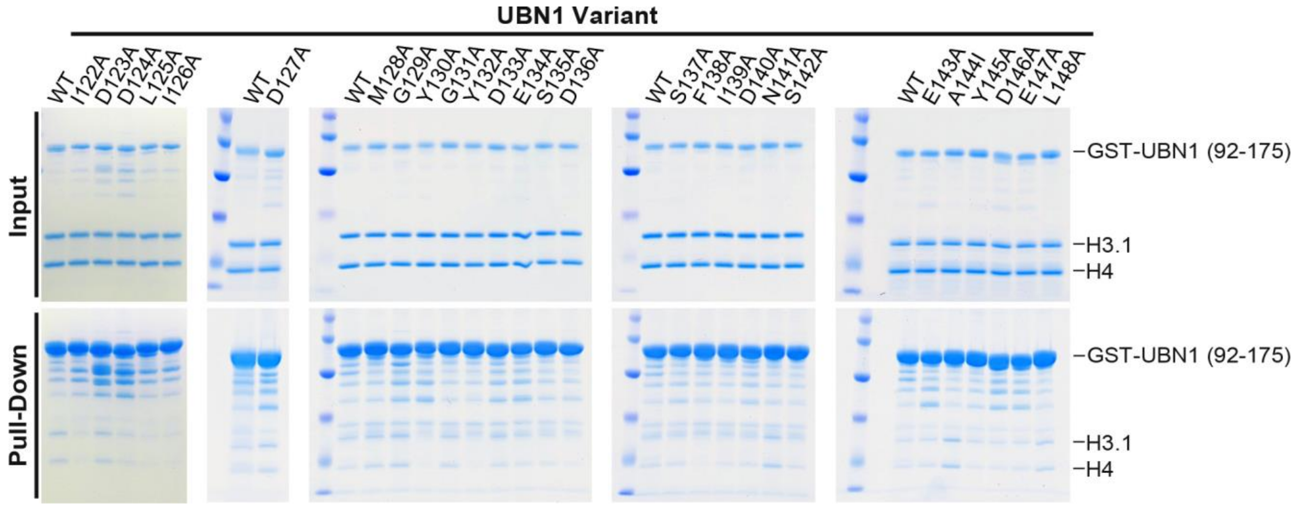

Supplementary Figure 4. GST pull-down to test the H3.1/H4 binding ability of alanine mutants of UBN1(92-175).

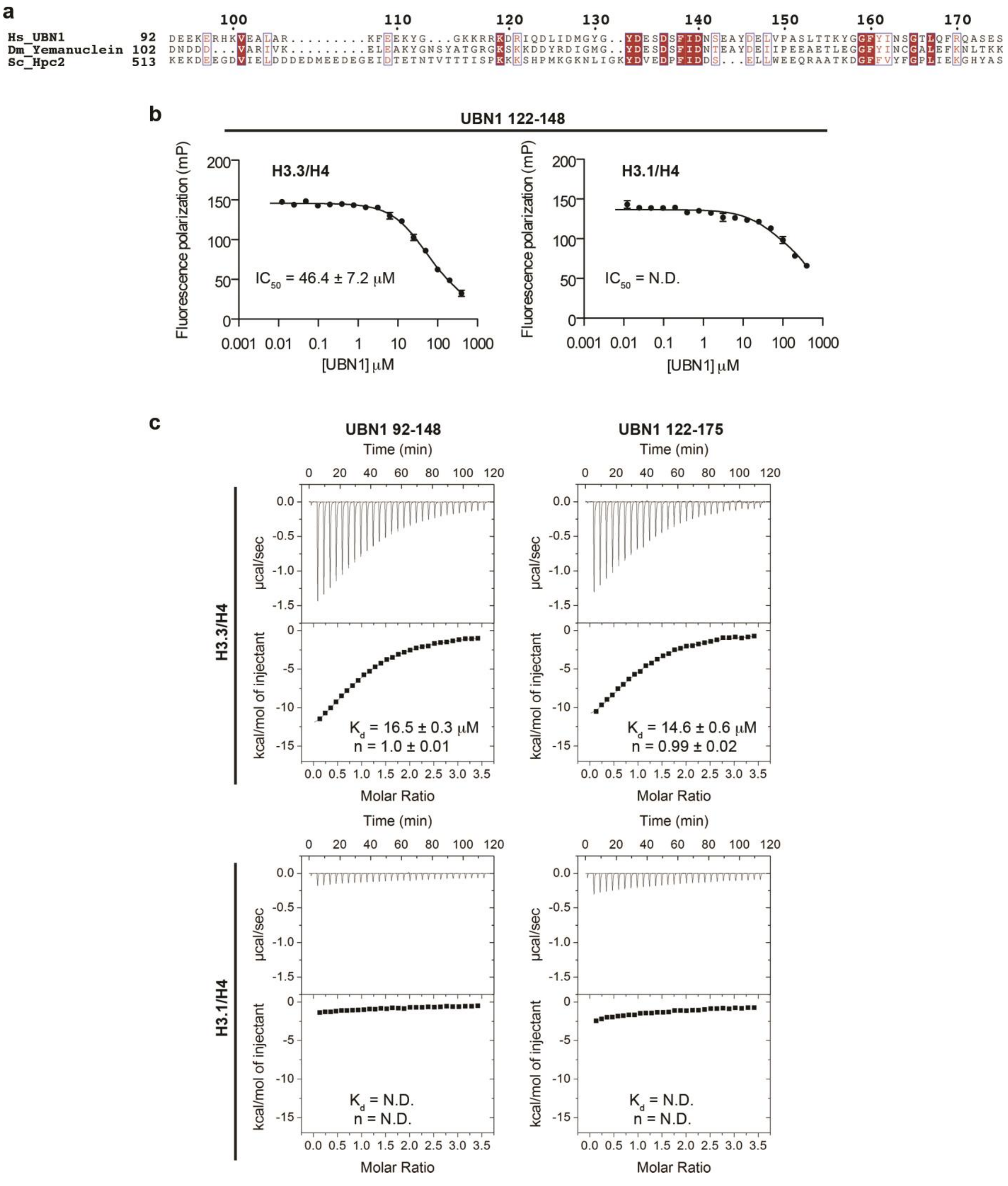

Supplementary Figure 5. Different HRD-containing UBN1 constructs binding with both H3.3/H4 and H3.1/H4. (a) Sequence alignment comparing residues in *Homo sapiens* UBN1, *Drosophila melanogaster* Yemanuclein, and *Saccharomyces cerevisiae* Hpc2, strictly conserved residues are highlighted in red. (b) Fluorescence polarization competition binding assay to quantify the binding of

UBN1(122-148) to both H3.3/H4 and H3.1/H4. (c) Isothermal titration calorimetry was used to confirm that both UBN1 (92-148) and (122-175) fragments specifically bind to an H3.3 containing H3/H4 complex, +/- values represent the standard error of the ITC fit using Origin 7.0.

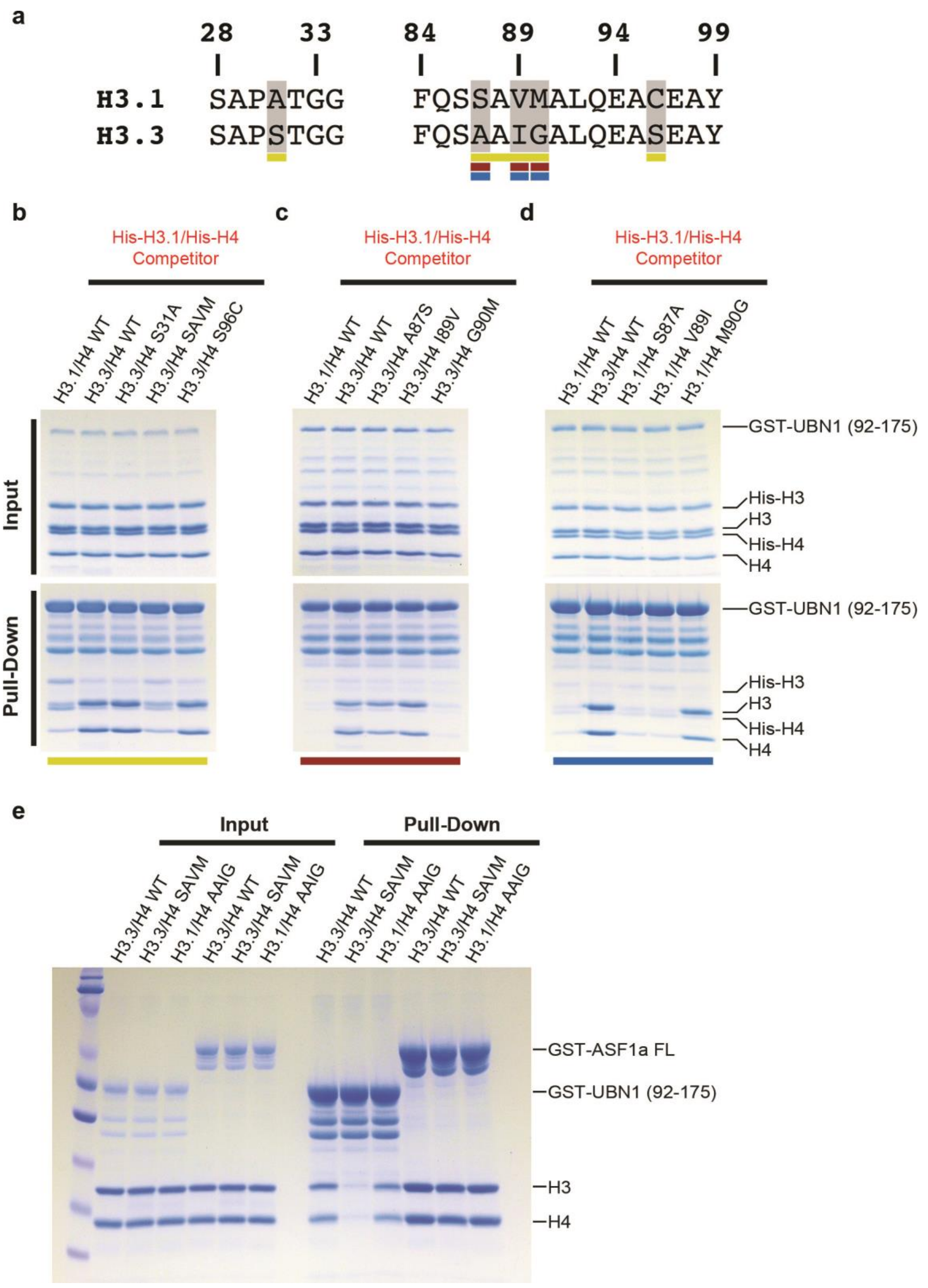

Supplementary Figure 6. Binding of UBN1 to H3.3 wild-type and mutants. (a) Sequence alignment highlighting the 5 differing residues between H3.3 and H3.1. Residues involved in pull-downs b, c and d are respectively underlined in yellow, red and blue. (b) GST pull-down indicating that the H3.3 residues AAIG mediate UBN1 specificity. (c) GST pull-down indicating that the H3.3 residue G90 is the crucial residue in the AAIG cluster responsible for UBN1 binding specificity. (d) Reverse GST pull-

down experiment that shows that the lack of specificity in UBN1 for H3.1 can be rescued with a M90G mutant.

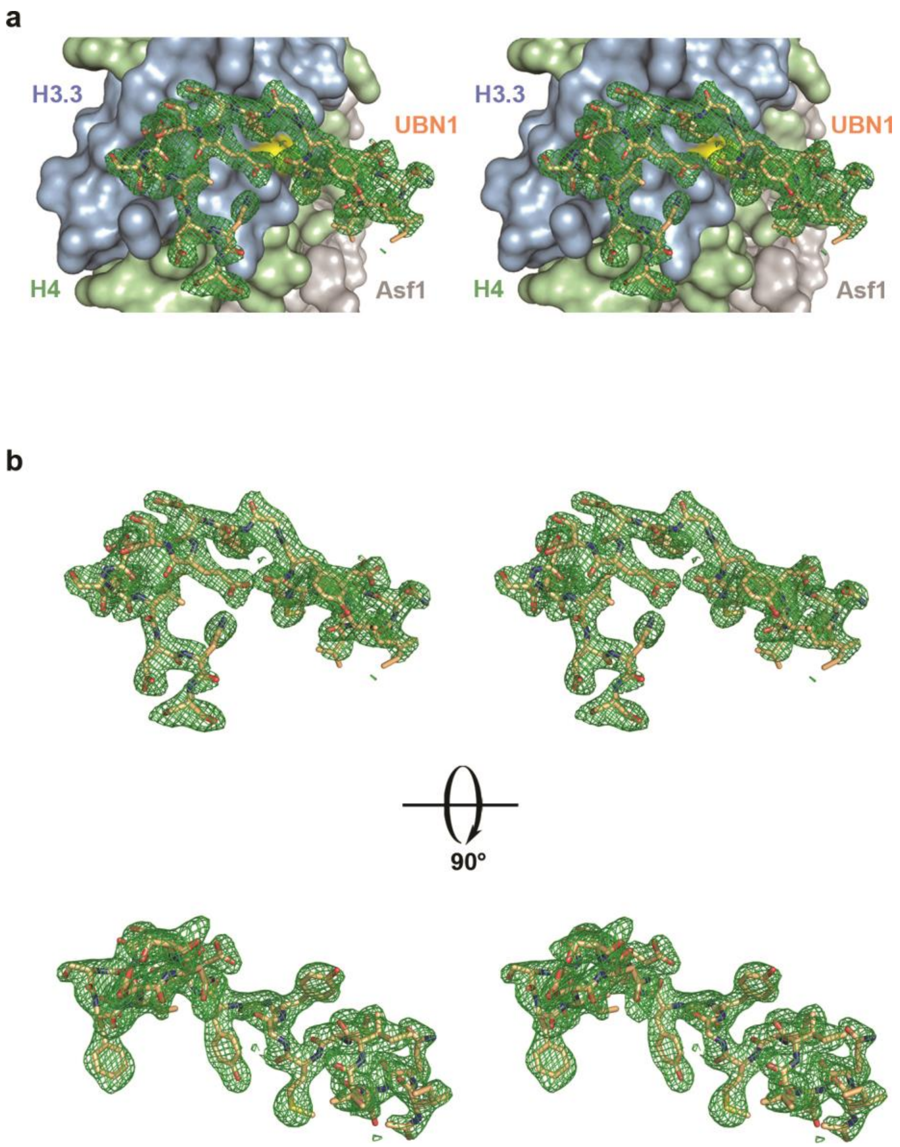

Supplementary Figure 7. Simulated annealing F_o - F_c omit map (contoured at 2σ) presented in stereo illustrating the high confidence of the UBN1 structural model. (a) Omit map was overlaid with the UBN1 structure (stick model) in the context of the bound H3.3/H4/Asf1 that is shown in surface representation in distinct colors. (b) View of the UBN1 omit map/structure overlay alone, with the same orientation as in (a) (top panel), or rotated 90 degree (bottom panel) for clarity of the various side chains.

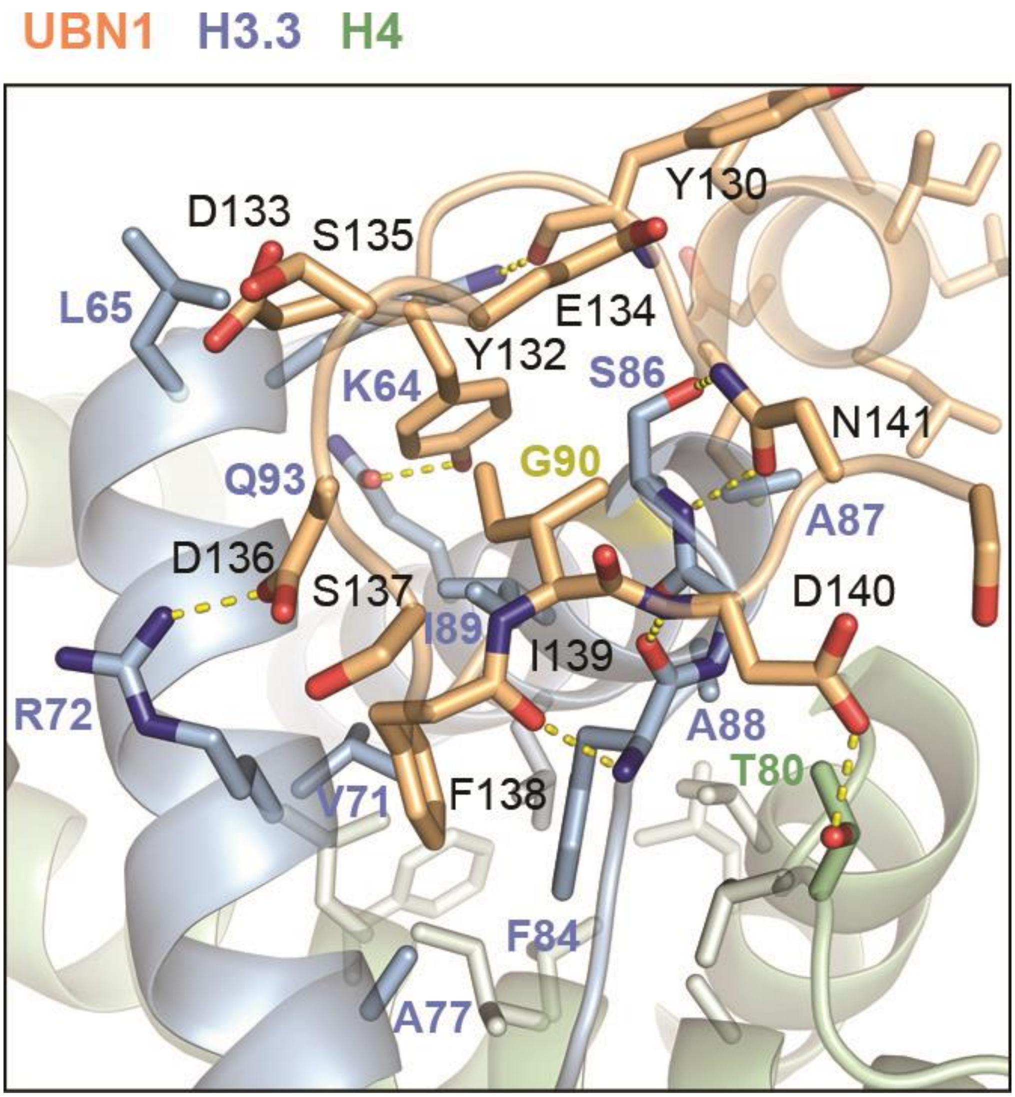

Supplementary Figure 8. Detailed view of the UBN1/H3.3/H4 interaction including UBN1 side chains E134, S135, and S137, which have no interaction with H3.3/H4.

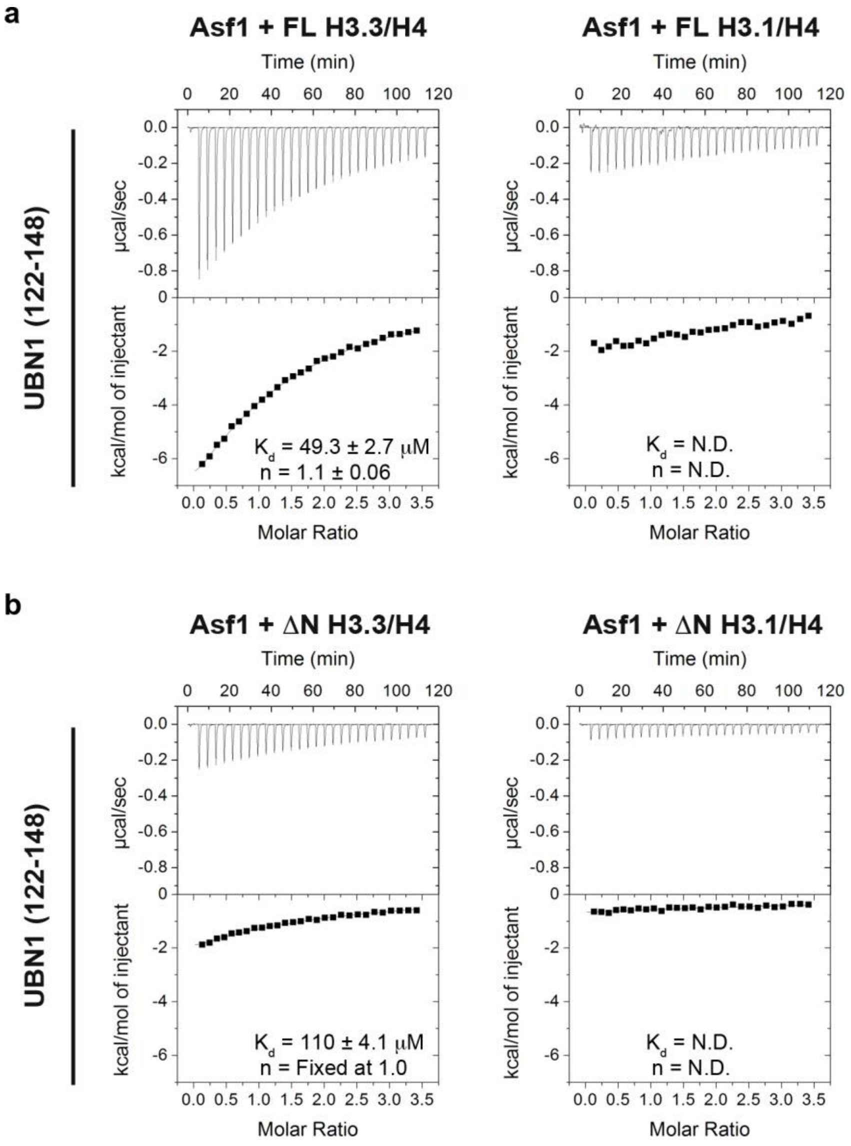

Supplementary Figure 9. ITC analysis of UBN1 binding to Asf1/H3/H4 complexes containing FL and N-terminally truncated histones. (a) ITC analysis of UBN1(122-148) interaction with FL H3.3/H4 or FL H3.1/H4 in complex with Asf1(1-169). (b) ITC analysis of UBN1(122-148) interaction with H3.3(60-135)/H4(20-102) or H3.1(60-135)/H4(20-102) in complex with Asf1(1-169), +/- values represent the standard error of the ITC fit using Origin 7.0.

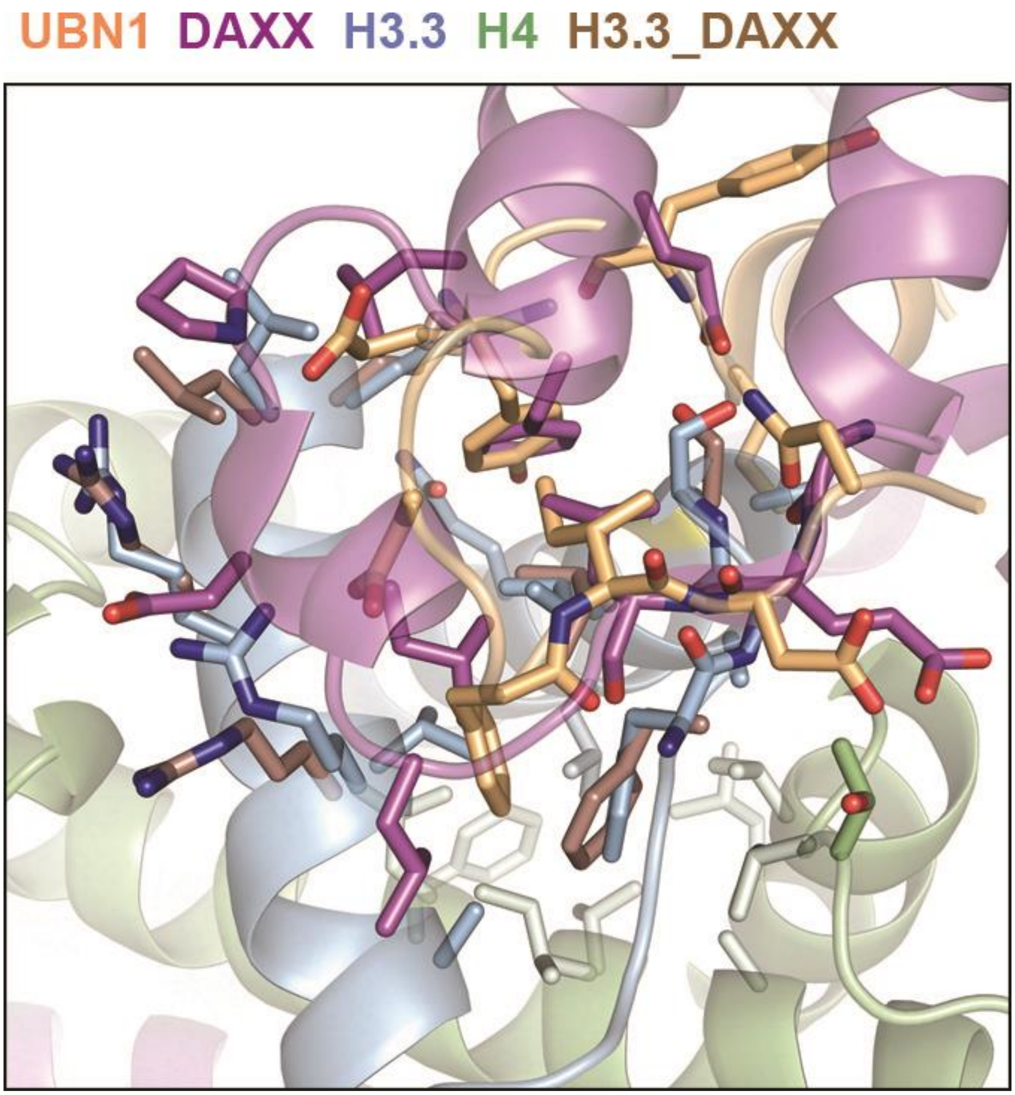

Supplementary Figure 10. Overlay of UBN1 and DAXX residues that contact the H3.3/H4 surface with close proximity to H3.3G G90, H3.3 residues from the DAXX/H3.3/H4 crystal structure that differ significantly from those in the UBN1/H3.3/H4/Asf1 structure are shown in brown.

Supplementary Table 1

H3.3 Residue	H4 Residue	UBN1 Residue	DAXX Residue
K64		Y130, Y132	Y222, L223
L65		D133	P218, L223
R69		X	D216
V71		F138	L215
R72		D136, F138	L212, L215
A77		F138	L212
F84		F138	L215
S86		N141	E225
I89		F138, I139	L210, L215
	T80	D140	E209
F84 - S86*		F138 - N141	K208 - L210

*Backbone contacts are formed within this group of residues

Supplementary Table 2

Primer Name	Primer Sequence (5'-3')
H4_N0_BamHI	CGCGGATCCATGTCTGGTCGTGGTAAA
H4_C102_XhoI	CCGCTCGAGTTAACCACCGAAACCGTAC
H3.3_N0_BamHI	CGCGGATCCATGGCTCGTACAAAGCAGACT
H3.3_C135_XhoI	CCGCTCGAGTTAAGCACGTTCTCCACGTATGCG
H3.1_N0_BamHI	CGCGGATCCATGGCCCGAACCAAGCAGACT
H3.1_C135_XhoI	CCGCTCGAGTTAGGCCCGCTCCCCGCGGATACG
H3.2_S96C_For	GCGCTGCAGGAGGCCTGCGAGGCCTACCTGGTG
H3.2_S96C_Rev	CACCAGGTAGGCCTCGCAGGCCTCCTGCAGCGC
H3.3_S32A_For	CAAGAGTGCGCCCGCTACTGGAGGGGT
H3.3_S32A_Rev	ACCCCTCCAGTAGCGGGCGCACTCTTG
H3.3_AAIGtoSAVM_For	AGATCTGCGCTTCCAGAGCTCAGCTGTCATGGCTTTGCAGGAGGCAAGTG
H3.3_AAIGtoSAVM_Rev	CACTTGCCTCCTGCAAAGCCATGACAGCTGAGCTCTGGAAGCGCAGATCT
H3.3_S96C_For	GCTTTGCAGGAGGCATGTGAGGCCTATCTGG
H3.3_S96C_Rev	CCAGATAGGCCTCACATGCCTCCTGCAAAGC
H3.3_A87S_For	GCGCTTCCAGAGCTCAGCTATCGGTGC
H3.3_A87S_Rev	GCACCGATAGCTGAGCTCTGGAAGCGC
H3.3_G90M_For	CTTCCAGAGCGCAGCTATCATGGCTTTGCAGGAGGCAAGTG
H3.3_G90M_Rev	CACTTGCCTCCTGCAAAGCCATGATAGCTGCGCTCTGGAAG
H3.1_S87A_For	CGCTTCCAGAGCGCGGCCGTGATGG
H3.1_S87A_Rev	CCATCACGGCCGCGCTCTGGAAGCG
H3.1_V90I_For	CCAGAGCTCGGCCATAATGGCGCTGCAGG
H3.1_V90I_Rev	CCTGCAGCGCCATTATGGCCGAGCTCTGG
H3.1_M90G_For	GAGCTCGGCCGTGGGGGCGCTGCAGGAG
H3.1_M90G_Rev	CTCCTGCAGCGCCCCCACGGCCGAGCTC
H3.1_SAVMtoAAIG_For	CTGCGCTTCCAGAGCGCGGCCATAGGGGCGCTGCAGGAGGC
H3.1_SAVMtoAAIG_Rev	GCCTCCTGCAGCGCCCCTATGGCCGCGCTCTGGAAGCGCAG
pST39_H3AAIG_For	ACCGACCTGCGCTTCCAGAGCGCCGCCATCGGTGCTCTGCAGGAGGCCAGCGA
pST39_H3AAIG_Rev	TCGCTGGCCTCCTGCAGAGCACCGATGGCGGCGCTCTGGAAGCGCAGGTCGGT
UBN1_N41_BamHI	CGCGGATCCGCTGCAGCAGCTGTTCGGATTAC
UBN1_N92_BamHI	CGCGGATCCAATGACGAAGAAAAGGAAAGGCAT
UBN1_N122_BamHI	CGCGGATCCATACAGGACTTGATCGATATGGGG
UBN1_C148_XhoI	CCGCTCGAGTTAAAGCTCATCATACGCCTCAGAGTT
UBN1_C175_XhoI	CCGCTCGAGTTAAGACTCTGATGCTTGTCTAAACTG
HIRA_N1_BamHI	CGCGGATCCATGAAGCTCCTGAAGCCGACCTGGGTC
HIRA_C405_XhoI	CCGCTCGAGTTACTGGTACTTGAGCATCTCAGGGTTCTC
HIRA_Untag_For	GCGCGGATCTCGGTCCGAAACCATGAAGCTCCTGAAGCCGACCTG
HIRA_Untag_Rev	CAGGTCGGCTTCAGGAGCTTCATGGTTTCGGACCGAGATCCGCGC
ASF1a_N1_BamHI	CGCGGATCCATGGCAAAGGTTCAGGTGAACAATGTAGTGG
ASF1a_C204_XhoI	CCGCTCGAGTTACATGCAGTCCATGTGGGATTCTAACATGAC
M1_UBN1_IQD_122-124_AAA_For	GTGGAAAGAAACGTAGAAAAGACCGAGCAGCGGCCTTGATCGATATGGGGTATGGTTATG
M1_UBN1_IQD_122-124_AAA_Rev	CATAACCATACCCCATATCGATCAAGGCCGCTGCTCGGTCTTTTCTACGTTTCTTTCCAC
M2_UBN1_LID_125-127_AAA_For	AACGTAGAAAAGACCGAATACAGGACGCGGCCGCTATGGGGTATGGTTATGATGAATCCG

M2_UBN1_LID_125-127_AAA_Rev	CGGATTCATCATAACCATACCCCATAGCGGCCGCGTCCTGTATTCGGTCTTTTCTACGTT
M3_UBN1_MGY_128-130_AAA_For	GAAAAGACCGAATACAGGACTTGATCGATGCGGCGGCTGGTTATGATGAATCCGACTCCT
M3_UBN1_MGY_128-130_AAA_Rev	AGGAGTCGGATTCATCATAACCAGCCGCCGCATCGATCAAGTCCTGTATTCGGTCTTTTC
M4_UBN1_GYD_131-133_AAA_For	GACTTGATCGATATGGGGTATGCTGCTGCTGAATCCGACTCCTTCATCGAT
M4_UBN1_GYD_131-133_AAA_Rev	ATCGATGAAGGAGTCGGATTCAGCAGCAGCATACCCCATATCGATCAAGTC
M5_UBN1_ESD_134-136_AAA_For	TATGGGGTATGGTTATGATGCAGCCGCCTCCTTCATCGATAACTCTG
M5_UBN1_ESD_134-136_AAA_Rev	CAGAGTTATCGATGAAGGAGGCGGCTGCATCATAACCATACCCCATA
M6_UBN1_SFI_137-139_AAA_For	GGGGTATGGTTATGATGAATCCGACGCCGCCGCCGATAACTCTGAGGCGTATGATGAG
M6_UBN1_SFI_137-139_AAA_Rev	CTCATCATACGCCTCAGAGTTATCGGCGGCGGCGTCGGATTCATCATAACCATACCCC
M7_UBN1_DNS_140-142_AAA_For	GATGAATCCGACTCCTTCATCGCTGCCGCTGAGGCGTATGATGAGCTTGT
M7_UBN1_DNS_140-142_AAA_Rev	ACAAGCTCATCATACGCCTCAGCGGCAGCGATGAAGGAGTCGGATTCATC
M8_UBN1_EAY_143-145_AAA_For	CCTTCATCGATAACTCTGCGGCGGCTGATGAGCTTGTTCCTGC
M8_UBN1_EAY_143-145_AAA_Rev	GCAGGAACAAGCTCATCAGCCGCCGCAGAGTTATCGATGAAGG
M9_UBN1_DEL_146-148_AAA_For	ATCGATAACTCTGAGGCGTATGCTGCGGCTGTTCCTGCTTCTTTGACTACG
M9_UBN1_DEL_146-148_AAA_Rev	CGTAGTCAAAGAAGCAGGAACAGCCGCAGCATACGCCTCAGAGTTATCGAT
M10_UBN1_VPA_149-151_AAA_For	GAGGCGTATGATGAGCTTGCTGCTGCTTCTTTGACTACGAA
M10_UBN1_VPA_149-151_AAA_Rev	TTCGTAGTCAAAGAAGCAGCAGCAAGCTCATCATACGCCTC
M11_UBN1_SLT_152-154_AAA_For	CGTATGATGAGCTTGTTCCTGCTGCTGCGGCTACGAAGTATGGAGGATTTTAC
M11_UBN1_SLT_152-154_AAA_Rev	GTAAAATCCTCCATACTTCGTAGCCGCAGCAGCAGGAACAAGCTCATCATACG
M12_UBN1_TKY_155-157_AAA_For	AGCTTGTTCCTGCTTCTTTGACTGCGGCGGCTGGAGGATTTTACATTAACTCGGG
M12_UBN1_TKY_155-157_AAA_Rev	CCCGAGTTAATGTAAAATCCTCCAGCCGCCGCAGTCAAAGAAGCAGGAACAAGCT
M13_UBN1_GGF_158-160_AAA_For	GCTTCTTTGACTACGAAGTATGCAGCAGCTTACATTAACTCGGGAACCCTG
M13_UBN1_GGF_158-160_AAA_Rev	CAGGGTTCCCGAGTTAATGTAAGCTGCTGCATACTTCGTAGTCAAAGAAGC
M14_UBN1_YIN_161-163_AAA_For	TTCTTTGACTACGAAGTATGGAGGATTTGCCGCTGCCTCGGGAACCCTGCAGTTTAG
M14_UBN1_YIN_161-163_AAA_Rev	CTAAACTGCAGGGTTCCCGAGGCAGCGGCAAATCCTCCATACTTCGTAGTCAAAGAA
M15_UBN1_SGT_164-166_AAA_For	GAAGTATGGAGGATTTTACATTAACGCGGCAGCCCTGCAGTTTAGACA
M15_UBN1_SGT_164-166_AAA_Rev	TGTCTAAACTGCAGGGCTGCCGCGTTAATGTAAAATCCTCCATACTTC
M16_UBN1_LQF_167-169_AAA_For	GAGGATTTTACATTAACTCGGGAACCGCGGCGGCTAGACAAGCATCAGAGTCTTAACTC
M16_UBN1_LQF_167-169_AAA_Rev	GAGTTAAGACTCTGATGCTTGTCTAGCCGCCGCGGTTCCCGAGTTAATGTAAAATCCTC
M17_UBN1_RQA_170-172_AAA_For	TACATTAACTCGGGAACCCTGCAGTTTGCAGCAGCATCAGAGTCTTAACTC
M17_UBN1_RQA_170-172_AAA_Rev	GAGTTAAGACTCTGATGCTGCTGCAAACTGCAGGGTTCCCGAGTTAATGTA
M18_UBN1_SES_173-175_AAA_For	CCTGCAGTTTAGACAAGCAGCAGCGGCTTAACTCGAGTCTGGTAA
M18_UBN1_SES_173-175_AAA_Rev	TTACCAGACTCGAGTTAAGCCGCTGCTGCTTGTCTAAACTGCAGG
UBN1_I122A_For	GTGGAAAGAAACGTAGAAAAGACCGAGCACAGGACTTGATCGA
UBN1_I122A_Rev	TCGATCAAGTCCTGTGCTCGGTCTTTTCTACGTTTCTTTCCAC
UBN1_Q123A_For	GAAACGTAGAAAAGACCGAATAGCGGACTTGATCGATATGGG
UBN1_Q123A_Rev	CCCATATCGATCAAGTCCGCTATTCGGTCTTTTCTACGTTTC
UBN1_D124A_For	GAAAAGACCGAATACAGGCCTTGATCGATATGGGGTA
UBN1_D124A_Rev	TACCCCATATCGATCAAGGCCTGTATTCGGTCTTTTC
UBN1_L125A_For	GAAAAGACCGAATACAGGACGCGATCGATATGGGGTATGGTT
UBN1_L125A_Rev	AACCATACCCCATATCGATCGCGTCCTGTATTCGGTCTTTTC
UBN1_I126A_For	AAGACCGAATACAGGACTTGGCCGATATGGGGTATGGTTATG
UBN1_I126A_Rev	CATAACCATACCCCATATCGGCCAAGTCCTGTATTCGGTCTT
UBN1_D127A_For	CGAATACAGGACTTGATCGCTATGGGGTATGGTTATGAT
UBN1_D127A_Rev	ATCATAACCATACCCCATAGCGATCAAGTCCTGTATTCG
UBN1_M128A_For	AAGACCGAATACAGGACTTGATCGATGCGGGGTATGGTTATG

UBN1_M128A_Rev	CATAACCATACCCCGCATCGATCAAGTCCTGTATTCGGTCTT
UBN1_G129A_For	CAGGACTTGATCGATATGGCGTATGGTTATGATGAATCC
UBN1_G129A_Rev	GGATTCATCATAACCATACGCCATATCGATCAAGTCCTG
UBN1_Y130A_For	GGACTTGATCGATATGGGGGCTGGTTATGATGAATCCGAC
UBN1_Y130A_Rev	GTCGGATTCATCATAACCAGCCCCCATATCGATCAAGTCC
UBN1_G131A_For	TGATCGATATGGGGTATGCTTATGATGAATCCGACTC
UBN1_G131A_Rev	GAGTCGGATTCATCATAAGCATACCCCATATCGATCA
UBN1_Y132A_For	CTTGATCGATATGGGGTATGGTGCTGATGAATCCGACTCCTTC
UBN1_Y132A_Rev	GAAGGAGTCGGATTCATCAGCACCATACCCCATATCGATCAAG
UBN1_D133A_For	GATATGGGGTATGGTTATGCTGAATCCGACTCCTTCATC
UBN1_D133A_Rev	GATGAAGGAGTCGGATTCAGCATAACCATACCCCATATC
UBN1_E134A_For	GGGGTATGGTTATGATGCATCCGACTCCTTCATCG
UBN1_E134A_Rev	CGATGAAGGAGTCGGATGCATCATAACCATACCCC
UBN1_S135A_For	CGATATGGGGTATGGTTATGATGAAGCCGACTCCTTCAT
UBN1_S135A_Rev	ATGAAGGAGTCGGCTTCATCATAACCATACCCCATATCG
UBN1_D136A_For	GTATGGTTATGATGAATCCGCCTCCTTCATCGATAACTCTG
UBN1_D136A_Rev	CAGAGTTATCGATGAAGGAGGCGGATTCATCATAACCATAC
UBN1_S137A_For	GGTTATGATGAATCCGACGCCTTCATCGATAACTCTG
UBN1_S137A_Rev	CAGAGTTATCGATGAAGGCGTCGGATTCATCATAACC
UBN1_F138A_For	TTATGATGAATCCGACTCCGCCATCGATAACTCTGAGGCG
UBN1_F138A_Rev	CGCCTCAGAGTTATCGATGGCGGAGTCGGATTCATCATAA
UBN1_I139A_For	TGATGAATCCGACTCCTTCGCCGATAACTCTGAGGCGTAT
UBN1_I139A_Rev	ATACGCCTCAGAGTTATCGGCGAAGGAGTCGGATTCATCA
UBN1_D140A_For	ATCCGACTCCTTCATCGCTAACTCTGAGGCGTATG
UBN1_D140A_Rev	CATACGCCTCAGAGTTAGCGATGAAGGAGTCGGAT
UBN1_N141A_For	GATGAATCCGACTCCTTCATCGATGCCTCTGAGGCGTATGA
UBN1_N141A_Rev	TCATACGCCTCAGAGGCATCGATGAAGGAGTCGGATTCATC
UBN1_S142A_For	ACTCCTTCATCGATAACGCTGAGGCGTATGATGAG
UBN1_S142A_Rev	CTCATCATACGCCTCAGCGTTATCGATGAAGGAGT
UBN1_E143A_For	CTTCATCGATAACTCTGCGGCGTATGATGAGCTTG
UBN1_E143A_Rev	CAAGCTCATCATACGCCGCAGAGTTATCGATGAAG
UBN1_A144I_For	TCATCGATAACTCTGAGATCTATGATGAGCTTGTTCC
UBN1_A144I_Rev	GGAACAAGCTCATCATAGATCTCAGAGTTATCGATGA
UBN1_Y145A_For	CATCGATAACTCTGAGGCGGCTGATGAGCTTGTTCCTGCT
UBN1_Y145A_Rev	AGCAGGAACAAGCTCATCAGCCGCCTCAGAGTTATCGATG
UBN1_D146A_For	TAACTCTGAGGCGTATGCTGAGCTTGTTCCTGCTT
UBN1_D146A_Rev	AAGCAGGAACAAGCTCAGCATACGCCTCAGAGTTA
UBN1_E147A_For	CTCTGAGGCGTATGATGCGCTTGTTCCTGCTTCTT
UBN1_E147A_Rev	AAGAAGCAGGAACAAGCGCATCATACGCCTCAGAG
UBN1_L148A_For	CTCTGAGGCGTATGATGAGGCTGTTCCTGCTTCTTTGACT
UBN1_L148A_Rev	AGTCAAAGAAGCAGGAACAGCCTCATCATACGCCTCAGAG
UBN1_FID_138-140_AAA_For	GGTATGGTTATGATGAATCCGACTCCGCCGCCGCTAACTCTGAGGCGTATGATGAGCTTG
UBN1_FID_138-140_AAA_Rev	CAAGCTCATCATACGCCTCAGAGTTAGCGGCGGCGGAGTCGGATTCATCATAACCATACC
